# Identifying biomarkers of neurodevelopmental and mental health outcomes in a prospective longitudinal cohort of South African children: design and feasibility of the Safe Passage BONO study

**DOI:** 10.1186/s40814-026-01790-1

**Published:** 2026-05-12

**Authors:** Priscilla E. Springer, Lucy T. Brink, Mandy Potter, Wendy Mackay, Petrusa C. Smit, Carlie du Plessis, Julia Koziel, Teresa Del Bianco, Bethany F. M. Oakley, Rianne Haartsen, Emily J H Jones, Marcus Munafò, Hein J. Odendaal, Declan G. Murphy, Eva Loth

**Affiliations:** 1 Department of Paediatrics and Child Health, Faculty of Medicine and Health Sciences, Stellenbosch University, Stellenbosch, South Africa; 2 Department of Obstetrics and Gynaecology, Faculty of Medicine and Health Sciences, Stellenbosch University, Stellenbosch, South Africa; 3 Department of Forensic and Neurodevelopmental Sciences, Institute of Psychiatry, Psychology and Neuroscience, King’s College London, London, UK; 4 Centre for Brain and Cognitive Development, Birkbeck, University of London, London, UK; 5 Present Address: School of Social Science and Profession, London Metropolitan University, London, UK; 6 MRC Integrative Epidemiology Unit, School of Psychological Sciences, University of Bristol, Bristol, UK; 7 Present Address: Vice Chancellor’s office, University of Bath, Bath, UK

## Abstract

**Background:**

Early adversities before and after birth can impact children’s cognitive and socioemotional development by altering critical brain maturational and functional processes. Some of these processes may be linked to later neurodevelopmental and/or mental health conditions. While most research is conducted in high-income countries, the majority of children live in low- and middle-income countries (LMICs) where they are more frequently exposed to poverty-related adversities. To address this gap, well-characterised longitudinal pregnancy cohorts in LMICs are needed to track trajectories of neurodevelopmental and mental health conditions in children exposed to cumulative environmental adversities. The Safe Passage Study (SPS) originally enrolled 7060 pregnant women from socioeconomically disadvantaged peri-urban communities in Cape Town, South Africa, to investigate the association between prenatal alcohol, multiple environmental risk factors and pregnancy outcome. The Safe Passage-Biomarkers of Neurodevelopmental Outcomes (BONO) study aims to follow up 2000 SPS children, aged 4–16 years, to assess the role of pre- and postnatal environmental factors in cognitive, neurodevelopmental and mental health outcomes. This report outlines the design and results of a feasibility study with 100 children, primarily aimed to confirm recruitment, assess participant retention, select measures and establish criteria for a deep-phenotyping visit.

**Methods:**

Between March and October 2019, 100 SPS children were screened during a “broad-phenotyping visit” for adverse childhood experiences and protective factors, autistic traits and socioemotional and behavioural symptoms, and they completed cognitive tests and eye-tracking and electroencephalography assessments to measure brain function. Criteria for a second “deep-phenotyping” visit were established based on autistic traits, internalising/externalising scores and/or cognitive difficulties, to assess children and their mothers in terms of clinical and neurocognitive profile.

**Results:**

Recruitment was adequate with a 96% retention rate for the deep-phenotyping visit. Feasibility study participants resembled the larger SPS cohort in most demographic and prenatal factors, except for higher prenatal depression and overcrowding indices. Most clinical and experimental measures were deemed suitable with minor modifications, and acquisition rates were high. The nature and length of visits were acceptable to families and testers. Threshold scores were adjusted to include 30% of participants for deep phenotyping.

**Conclusions:**

The feasibility study fulfilled progression criteria for the planned multimodal study.

**Supplementary Information:**

The online version contains supplementary material available at 10.1186/s40814-026-01790-1.

## Key messages regarding feasibility

1. What uncertainties existed regarding the feasibility
2. What are the key feasibility findings?
3. What are the implications of the feasibility findings for the design of the main study?
  Uncertainties included
    Recruitment and retention of participants for both study visits
    The suitability of applying clinical/experimental measures to a low-resource setting which were acceptable to participants and selection of emotional/behavioural and/or cognitive criteria that would qualify participants for a deep phenotyping phase
  Key feasibility findings
    Recruitment and retention were successful, but participants who independently sought study enrolment differed from those initially recruited by the researchers.
    Specific clinical/experimental measures were chosen after minor adaptations and acquisition rates were high. Thresholds (cut-off scores) for key measures were adjusted for deep phenotyping participant selection.
  Implications for design of main study
    Participant selection was adjusted to reduce recruitment bias.
    Clinical measures needed adaptations when used in a different cultural context. Selected thresholds for deep phenotyping phase restricted participant numbers to align with available resources.

## Background

Early-life adversities occurring before and after birth can have a profound impact on children’s social, cognitive and emotional development by altering critical brain maturation and functions, as well as interacting physiological, immune and endocrine processes [1]. These neuropsychological processes can in turn have long-term effects on life outcomes (health, education, employment). They are also, to differing degrees, implicated in many neurodevelopmental and mental health conditions. In low- and middle-income countries (LMICs), children are more frequently exposed to pre- and postnatal adversities that are directly or indirectly linked to poverty. For example, South Africa has high rates of malnutrition, recreational drug and alcohol use and smoking during pregnancy, intimate partner violence, maternal anxiety and depression, child abuse and neglect and community violence [2–5].

When occurring prenatally, these adversities can affect foetal brain development by modulating epigenetic regulation and placental function, with long-term effects on microglial activation, hypothalamic–pituitary–adrenal axis (HPA) function, stress reactivity, upregulation of neuroinflammatory processes and hormone levels in childhood and adolescence [1, 6, 7]. These interconnected factors also contribute to subsequent perinatal risk, such as premature birth, low birthweight and obstetric complications. After birth, some of these risk factors translate or contribute to “adverse childhood experiences” (ACEs) across childhood and adolescence. Each ACE and their combination can affect children’s psychological development through psychosocial mechanisms as well as chronic stress [8]. For example, in the USA, ACEs were strongly associated with the presence of conduct problems, depression and substance abuse and moderately associated with some neurodevelopmental conditions, e.g. autism and attention-deficit hyperactivity disorder [9].

Recent estimates suggest that nearly 40% of children living in LMICs may be at risk of not reaching their developmental potential, leading to academic and economic underachievement [10]. Although over 80% of children are born in LMICs, the vast majority of research on neurodevelopmental and mental health conditions is conducted in high-income countries (HIC) [11]. This raises the need to better understand the presentation and progression of neurodevelopmental and mental health conditions in children exposed to cumulative environmental and social adversities in LMICs.

In many LMICs, including South Africa, precise prevalence estimates of neurodevelopmental and mental health conditions are lacking, partly due to limited access to diagnostic tools and services as well as varying perceptions of mental health [12]. The number of children living with neurodevelopmental conditions and other childhood disabilities in sub-Saharan Africa has increased, aligned with decreasing infant mortality rates since 1990 and increased child survival [13]. Prevalence estimates for neurodevelopmental and mental health conditions in LMICs are often extrapolated from those in HIC [11]. Here, 10–15% of children are estimated to have a neurodevelopmental condition; this umbrella includes autism spectrum disorder (henceforth autism) with global prevalence estimates of 0.7–3%, attention-deficit hyperactivity disorder (ADHD) (5–11%), intellectual disabilities (0.63%), language disorders (1–3%), motor disabilities (0.74–17%) and others [14]. Mental health conditions are estimated to affect around 20% of the population globally and include anxiety, depression, post-traumatic stress disorder, schizophrenia, bipolar disorder, obsessive compulsive disorder, substance use disorders and personality disorders [15].

Over the past decade, research has highlighted both substantial *diversity* within specific neurodevelopmental and mental health conditions as well as frequent *overlap* between conditions [16]. This means that neurodevelopmental and mental health features not only frequently co-occur in one individual (e.g. 28–53% of autistic people have ADHD [17], 42% an anxiety disorder [18] and 37% depression [19]) but that these conditions also often co-occur in the same families. Both common pleiotropic genetic factors, as well as psychosocial factors (parental style, intergenerational transmission), gene-environmental correlations and interactions are likely to play a role. As a result, a categorical diagnosis alone is limited in predicting a child (or person’s) developmental trajectory, their therapeutic needs and the likely efficacy of particular therapies or in making inferences regarding the underlying cause and mechanisms in a specific individual [16]. Precision medicine is an increasingly influential research approach in psychiatry, originally developed by internal medicine, with the goal to enhance clinical predictions and provide better targeted support by matching mechanism-based interventions to individual needs and biological profiles using biomarkers. One example of this approach is the EU-AIMS/AIMS-2-TRIALS consortia, which aim to develop precision medicine for autistic individuals and people with related neurodevelopmental conditions [20, 21]. The biomarker programme of AIMS-2-TRIALS adopts a longitudinal, transdiagnostic and multidisciplinary design where, in linked studies, participants are followed from before birth to adulthood in order to identify mechanisms and markers underlying the development and prognosis of clinical features or behavioural profiles. However, these studies are being conducted in European countries with a large proportion of study participants from relatively affluent backgrounds [22]. Hence, it is unclear to what extent mechanisms and markers identified in these studies may generalise to other cultural and economic settings. This is a critical limitation because, as indicated above, environmental factors and (chronic) adverse experiences are known to impact brain and psychological development, and these exposures are more common in some LMIC settings [23]. Moreover, it is likely that social-cultural factors, including awareness of neurodevelopmental/mental health conditions and stigma, impact the development, presentation or progression of these conditions and quality of life of neurodivergent people and people with mental health conditions [24, 25].

The current study aims to increase our understanding of the mechanisms by which social and environmental risk and resilience factors impact on brain, cognitive and social-emotional development and the development and progression of neurodevelopmental and mental health conditions. It capitalises on the South African Safe Passage Study (SPS), a prospective longitudinal study of 7060 pregnant women, predominantly of mixed ancestry, and their infants from two lower socioeconomic residential areas near Cape Town [26]. In LMICs in general, and South Africa in particular, only a few pregnancy or birth cohort studies exist that assess both biological and social factors. For example, the birth to 30 cohort, a prospective longitudinal study, enrolled over 3000 children prenatally in 1990 in Johannesburg [27, 28], while the Siyakhula study enrolled 1536 children, aged 7–11 years from rural KwaZulu-Natal, and assessed the children’s cognition, emotion and behaviour using a cross-cultural test battery [29]. The ongoing Drakenstein Child Health Study includes a peri-urban cohort (1000 participants) of similar socio-economic status to the SPS, which is also enrolled prenatally [30] but with a main focus on maternal mental health and early childhood physical health and development. To our knowledge, the SPS cohort is the only longitudinal South African pregnancy cohort with contemporaneous documentation of prenatal exposures and detailed monitoring of foetal and neonatal physiology.

The SPS study was originally undertaken to investigate the role of prenatal alcohol exposure in pregnancy outcomes, including the occurrence of stillbirth and sudden infant death syndrome. A total of 7060 pregnant women were enrolled in the South African limb of the study between 2007 and 2015 with infants followed up until 1 year of age. Maternal and foetal assessments were conducted at enrolment and during up to three prenatal clinical visits, while mother–child dyads were seen postnatally at newborn and 1-month and 1-year visits. Infant age at the three postnatal visits were adjusted for prematurity. This comprehensive data set includes information on maternal physical and mental health, placental function, foetal physiology and growth and newborn and infant development [26]. Four embedded follow-up studies reassessed 615 children between age 3–4 years in terms of socioemotional development [31], 500 children at 4 years in terms of children’s social and cognitive development [32], 500 children at 6 years to examine child health [33] and 500 at 5–6 years in terms of brain development, including neuroimaging [34], and recruitment was adequate in all cases.

The primary aim of the BONO study is to identify social and biological markers and mechanisms involved in the development and/or presentation of neurodevelopmental and mental health conditions by reassessing SPS children between the ages of 4–16 years. The design comprises two distinct phases: A broad-phenotyping phase (BP) aimed at screening 2000 children in terms of adverse (and protective/promotive) childhood experiences, social and cognitive development, externalising/internalising behaviours and brain function. A subset of children will then be selected for a deep-phenotyping phase (DP) based on either “high versus low” autistic traits, internalising/externalising scores, or cognitive abilities (i.e. meeting pre-specified measurement thresholds for difficulties on specific measures) or chosen randomly to serve as controls.

Secondly, we aim to apply cognitive and brain functional markers and mechanisms identified in the European AIMS-2-TRIALS studies to the SPS cohort. The majority of cognitive, behavioural and mental health measures were developed and validated in the Global North, as measures standardised in LMICs and sensitive to the South African cultural and socioeconomic context are largely unavailable [35–37].

Hence, given the scope of BONO, we carried out a feasibility study to understand the appropriateness of applying our planned study design and measures to this South African cohort. We based our objectives on those listed by Orsmond and Cohn (2015) and the STROBE guidelines when reporting outcomes of feasibility studies [38, 39]. We planned to (1) determine ease of participant recruitment/retention and resulting sample characteristics, in order to determine representability of BONO against the original SPS cohort; (2) evaluate comprehensibility, suitability and cultural appropriateness of experimental and clinical/behavioural outcome measures in order to make final protocol selection; (3) assess the burden of the proposed protocol on testers and families and adjust accordingly; (4) establish cut-off criteria for the deep-phenotyping phase to include a maximum of 33% of cohort; (5) ensure there were adequate resources to manage and implement the study; and (6) explore referral pathways to confirm that there was service capacity for children detected by screening tests who required further evaluation by health, social and educational services.

## Methods

### Study design

The feasibility study was a cross-sectional, observational study designed to provide preliminary data, to allow for final selection, translation and adaptation (where relevant/appropriate) of measures, and to inform standard operating procedures (SOP) for the full phase of the BONO longitudinal cohort study.

### Participants

Inclusion criteria for the feasibility study were mothers with children, aged between 3 and 12 years, previously enrolled in the SPS, who had consented to be contacted for future studies. Children who had experienced a change in caregiver over the preceding 6 months were excluded to maximise validity of caregiver report measures. The children were divided into four age bands: 3–4.9 years, 5–6.9 years, 7–8.9 years and 9–11.9 years.

Initially, a total of 46 invitation letters were hand-delivered by the study driver on two occasions. Prospective participants were provided with an overview of the study, which took place at Tygerberg Academic Hospital (SPS research site). Those interested were asked to phone the SPS unit, confirm contact details, were offered further information and were placed on the database. The research coordinator then phoned the participants to arrange the assessment date.

One hundred children and their mothers were enrolled between 27th March and 29th October 2019 and included random selection of at least 10 children in each age band with approximately equal numbers of boys (45%) and girls.

### Procedure

Participants were transported by the study driver from the community to the SPS research site on two separate occasions within a maximum time frame of 4 weeks. The two visits broadly followed the intended design of the planned BONO study (broad/deep phenotyping), with the following exceptions: in contrast to the main study design, all mother–child pairs returned for the second visit, and neurophysiological assessments were performed at both visits, with the goal to select the best-performing tasks.

### Clinical and behavioural measures

During the “broad-phenotyping” phase, the mothers were asked to complete a series of questionnaires assessing quantitative features related to a range of neurodevelopmental or mental health conditions, including their child’s autistic traits and social communication (Social Communication Questionnaire) [40], repetitive behaviours (Childhood Routines Inventory-Revised) [41, 42] and sensory processing (Sensory Experience Questionnaire) [43], internalising (anxiety, depression, somatisation) and externalising behaviours (aggression, hostility, hyperactivity) (Strength and Difficulties Questionnaire) [44], child temperament (Child Behaviour Questionnaire) [45] and schizotypy and psychotic traits (Childhood Oxford-Liverpool Inventory of Feelings and Experiences—CO-LIFE) [46]. One study goal was to define cut-off scores to identify a subset of participants for “deep phenotyping” on three measures: the Social Communication Questionnaire (SCQ), the Strength and Difficulties Questionnaire (SDQ) and the Wechsler Abbreviated Scales of Intelligence-second edition (WASI), a standardised measure of verbal and non-verbal intelligence [38].

During the feasibility study, we also introduced the risk and protective factors questionnaire, which assesses a range of adverse childhood experiences (based on the WHO Adverse Childhood Experiences) [47], including neglect, abuse, family dysfunction, additional community-relevant adversities (e.g. neighbourhood violence) and protective factors (e.g. supportive parental relationships).

During the deep-phenotyping visit, caregivers completed similar questionnaires assessing autistic traits (Social Responsiveness Scale-2), internalising externalising behaviours and schizotypy (SDQ, O-LIFE) about themselves, as well as questionnaires about their child’s temperament (Child Behaviour Questionnaire) (for the specific measures used, please see Tables 1 and 2). These questionnaires are described in more detail in Appendix 1.

**Table 1:** Overview of parent and child measures during broad phenotyping (Visit 1)

	Domain	Measure
	Child health information	Weight, height and head circumference
Parent	Past medical/family history	Demographics and medical history questionnaire
	Autistic traits/behaviours	Social Communication Questionnaire (SCQ)
	Mental health	Strengths and Difficulties Questionnaires (SDQ) Childhood Oxford-Liverpool Inventory of Feelings and Experiences (CO-LIFE) schizotypy subscale
	Childhood adversities and protective factors	Risk and Protective Factor Questionnaire 24 items (stressful life events and protective support)
	Total time (parent)	**Estimated time for parents: 60 min** (plus breaks)
Child	Non-verbal cognitive ability	*Leiter International Performance Scale-3rd edition, only fluid intelligence subscales
	Language development and cognition	Age 3–6 years: Expressive/receptive language subtests of the Mullen Scales of Early Learning (MSEL) Age 6–12 years (or in younger child if ceiling on MSEL is reached): Wechsler Abbreviated Scales of Intelligence-second edition (WASI) vocabulary/similarity subscales/matrix reasoning/block design
	Tablet tasks	Tasks evaluating inhibitory control, sustained attention, reward learning, emotion recognition, theory of mind
	EEG tasks	Event-related potential task, three resting-state conditions, measurement of sensory processing efficiency
	Total time (child)	**Estimated time for child: 85 min** (plus breaks)

**Table 2:** Overview of parent and child measures during deep phenotyping (Visit 2)

	Domain	Measure
Parent	Autism diagnosis	*Childhood Autism Rating Scale-revised (CARS)
	Repetitive behaviours and restricted interests in child	• Childhood Routines Inventory-Revised (CRI-R)
	Sensory atypicalities in child	• Sensory Experience Questionnaire (SEQ)
	Child temperament	*Child Behaviour Questionnaire (CBQ)
	Adult autistic symptoms Adult mental health symptoms	*Social Responsiveness Scale-2nd Edition (SRS) • Strengths and Difficulties Questionnaire (adult self-report version) (SDQ) • Oxford-Liverpool Inventory of Feelings and Experiences O-LIFE (schizotypy subscale) (O-Life)
	Maternal cognition	*Leiter-III (fluid IQ subscales) Or Wechsler Abbreviated Scales of Intelligence-second edition (WASI)
	Total time (parent)	**Estimated time for parent: 135 min** (plus breaks)
Child	Tablet tasks	Tasks assessing decision-making under uncertainty, preference for social novelty, sensory processing (frequency discrimination)
	Eye tracking	Spontaneous social attention, static and dynamic social scenes
	EEG*	Event-related potential task, three resting-state conditions, measurement sensory processing efficiency
	Total time (child)	**Estimated time for child: 70 min** (plus breaks)

*Measures which were omitted or exchanged in main study

Questionnaires were read to the participants by a research worker rather than self-administered, as specific questions on autism or mental health might be misinterpreted or require further explanation and/or some mothers had limited literacy [48]. Measures were translated by external and in-house services from English to Afrikaans applying the standard forward and backward translation to address the two predominant languages spoken in this community [48].

Children and their mothers were assessed in parallel.

After each testing session, the research workers carried out a more in-depth individual debriefing interview with the mothers, to ask about their experience taking part in the study and obtain their views on feasibility, comprehensibility and acceptability of the standardised measuring instruments.

All testers received initial training and supervision from the lead developmental psychologist (E. L.) with respect to cognitive and behavioural assessments and formal instruction on autism. Feedback sessions were held regularly to answer questions on all measures. Inter-rater reliability was established on the cognitive assessments.

Before the study start, we informed the community psychiatric mental health and social services about the study, in order to confirm adequate capacity and met with social workers from the Tygerberg Hospital Social Worker Department to establish a direct referral pathway for adult or child participants requiring further diagnostic evaluation.

### Electroencephalograpy (EEG)

The portable 20-channel Enobio electroencephalogram was introduced in this protocol as a noninvasive technique to objectively measure functional brain responses to social and sensory paradigms using ad hoc stimuli, e.g. social scenes and auditory odd-ball, and “at rest”, which complement psychological and cognitive assessments. The EEG battery consisted of 5 tasks with a total duration of 30 min, during which changes in brain potential with high temporal resolution were measured through electrodes at 19 locations across the head, and 1 light sensor to correct for timing delays. The face early receptor potential (ERP) is an event-related potential task that examined the face inversion effect—a quicker and more negative early inflection of brain potentials in response to upright human faces compared to inverted faces and control stimuli, a phenomenon that strengthens across childhood [49]. Additionally, we measured brain oscillations across different frequency bands, while participants watched naturalistic videos of women singing nursery rhymes or spinning toys (social/nonsocial videos task) and a picture of a fixation cross during a traditional eyes-open resting state [50, 51]. Furthermore, an auditory steady-state task and an auditory oddball task were included to measure sensory-processing efficiency [52, 53]. The paradigm included remote eye tracking to monitor the participant’s gaze on the screen, making all tasks gaze contingent and adapting the pace of the session to the participant’s attention span [54].

### Touch screen tasks

We piloted beta versions of a suite of touch screen tests (version 0.6–0.8) that were being developed in a separate project by the study team, with the goal to assess six biobehavioural domains implicated in neurodevelopmental and mental health conditions; this included social, emotional, reward, executive function, un/predictability and sensory processes. During this feasibility study, touch screen tests, administered on Lenovo tablets, assessing inhibitory control and sustained attention, social and non-social reward processing, emotion recognition and theory of mind, were piloted in a subsample of children. Scripts/instructions were translated into Afrikaans in-house.

Total time of each assessment visit was originally estimated to be a maximum of 150 min, excluding flexible breaks when needed. Participants received a supermarket voucher (250 ZAR or approximately US $17) as compensation for their time, and each child received a toy or stationery.

### Progression criteria

Progression criteria included firstly over 90% participant retention for both broad and deep phenotype visits and secondly the final selection of clinical measures with at least 90% acquisition rate reflecting acceptability to participants. Progression criteria were agreed on with stakeholders (researchers and AIMS-2 Steering Committee) to determine the success of the feasibility study with subsequent progression to main study.

### Statistical analysis

The feasibility study sample size calculation was based primarily on estimating 90th percentile cut-off scores for the behavioural questionnaire (SDQ) in the broad phenotype visit. This was to ensure the deep phenotype participant numbers aligned with available resources.

The secondary aims of the feasibility study were to refine the methods and measures.

The estimation of the probability of a success in a Bernoulli trial (for large populations) was used to estimate the probability (p) of success from the feasibility study sample. A sample size of 92 should be sufficient to ensure that a 95% confidence interval for p will have an error rate of 10%. Thus, the eventual sample size of 100 should leave sufficient provision for any incomplete assessments.

This sample size was also deemed sufficient to determine suitability of measure and acceptability of the protocol to participants and testers and make adaptations, i.e. calculate descriptive statistics (means/medians, standard deviations, ranges) to interrogate the distribution of scores in order to ascertain potential floor (or ceiling) effects of measures, as an indicator of suitability for use in a community (as opposed to clinical) sample and for initial comparison to expectations from standardised scores (where available and with the caveat that those were based on HIC studies).

We also performed correlation analyses to examine the relationship between measures tapping into the same or related constructs (notably autism trait measures) and to explore the relationship between mother–child performances on selected measures. The goal of these analyses was to detect performance patterns indicative of unsuitability of a measure and to inform decisions on measures to be carried forward to the main study. Pairwise comparisons were performed to interrogate changes in demographics between the time point of the original Safe Passage inception study and the current feasibility study (children between 3 and 12 years). Between-group comparisons were carried out to examine potential differences between feasibility study participants and the total SPS cohort, as well as study participants responding to invitations versus those who contacted the team out of their own volition (self-referred).

### Ethical considerations

Ethical approval was obtained from the Health Research Ethics Committee, Stellenbosch University (N18/08/090), and permission from Tygerberg Academic Hospital. It was anticipated that caregivers might experience psychological stress associated with questionnaires assessing clinical features or anxiety should their child need referral for diagnostic evaluation. The research nurse consulted with the developmental paediatrician regarding any unresolved matters. When medical or psychological conditions requiring further intervention were identified by research staff, the participants were referred to the appropriate health, social or educational services.

## Results

### Recruitment and characteristics of feasibility study participants

It is important to note that all analyses beyond the feasibility measures are purely exploratory. Twenty-six out of 46 participants who had received hand-delivered letters responded within 48 hours; in all, 26 participants agreed to the study, 16 did not respond, 1 was deceased, 2 had relocated and 1 was interested but unable to attend within the time frame. A further 74 participants heard about the study from the other participants, phoned the research unit or recognised and stopped the driver to give their contact details. Some caregivers (16 participants) heard from a researcher recruiting for another study at the local antenatal clinic, where SPS mothers were sitting in the waiting room. The recruitment of 100 participants was completed within 3 months. Those who could not be accommodated in the feasibility study were placed on a waiting list for the main study. The participants for the feasibility study were selected to include 10 children in each age category from 3 to 12 years.

One hundred participants attended the broad phenotyping visit, of which 96 participants also attended the second deep-phenotyping visit. Two participants were unable to attend their scheduled deep-phenotyping visit due to personal reasons, one due to withdrawal (no reason given) and one due to relocation. The participant sociodemographic information is shown in Tables 3 and 4. Overall, 59 children were in formal education (schoolgoing), 13 attended pre-school, 15 children were in informal day-care centres and 13 pre-school-aged children spent their day at home.

**Table 3:** Children’s home language versus school (language of instruction)

**Child variable**	N/total(%)
Male sex	55/100 (55)
Home language Afrikaans	78/100 (78)
Language of instruction Afrikaans	53/91 (58)
Language of instruction English	37/91 (40.7)
Home language—English	12/100 (12)
Home language—bilingual	10/100 (10)
Language instruction—bilingual	3/91 (3.3)

**Table 4:** Comparison of maternal demographic characteristics at prenatal and feasibility study visits (*N* = 100)

	**Antenatal period**				**Feasibility study period**			
**Variable**	**Mean**	**SD**	**Median**	**Range**	**Mean**	**SD**	**Median**	**Range**
Crowding index (people/room)	1.8	0.9	1.5	0.6–5.5	0.73	0.31	0.67	0.2–2.0
Gravidity	2.5	1.5	2.0	1–7	3.3	1.6	3	1–9
Household income per month (ZAR)	718	425	714	83–2000	5883	4614	4700	400–24,000
	**n/N (%)**				**n/N (%)**			
Employed	22/94 (23.4)				34/100 (34.0)			
Married	19/100 (19.0)				41/100 (41.0)			
Single or divorced	4/100 (4.0)				34/100 (34.0)			
Partners living together	27/100 (27.0)				21/100 (21.0)			
*Child support grant	28/100 (28.0)				87/100 (87.0)			
Formal housing	51/100 (51.0)				59/100 (59.0)			
Informal/backyard dwelling	26/100 (26.0)				34/100 (34.0)			
Apartment	22/100 (22.0)				7/100 (7.0)			

*The child support grant is a government grant of ZAR500 per month ($28) awarded to any primary caregiver, including South African citizens, permanent residents or refugees whose income falls below R4500 ($247) per month (2019)

The children’s home language was predominantly Afrikaans (78%), followed by English (12%) and bilingual (10%), i.e. English and Afrikaans. However, only 58% (*N* = 53) of school and pre-school-going children were educated in Afrikaans, while 42% (*N* = 38) had English as the language of instruction. This may partly be attributed to the general impression that English is viewed as an international language and could lead to better employment opportunities.

Ten children (8 boys and 2 girls) were stunted (heights < − 2 Z-score), 12 children (10 boys and 2 girls) were underweight (weights < − 2 Z-score) while 2 children (1 boy) were overweight (weights > + 2 Z-score). These findings are consistent with other South African studies [55, 56].

Table 4 illustrates how the participants’ domestic situations had changed between the prenatal inception study and the feasibility study (average of 8 years between visits). By the time of the feasibility study, a greater proportion of mothers were married, single or divorced and had some form of employment. We did not document the number of mothers who were single with partners living elsewhere, although this constituted the most common living arrangement in the antenatal period. More feasibility study participants now lived in free-standing homes than apartments, and they reported less crowded living conditions than during pregnancy (see Table 4).

We detected a significant difference between the participants who responded to the invitation letters versus those who contacted the unit independently in terms of demographic and lifestyle characteristics (Table 5). Compared to the invited participants (*N* = 26), this latter “self-referred” group (*N* = 74) had on average fewer years of education and during pregnancy had reported lower mean monthly income, higher antenatal alcohol intake, more binge episodes and a greater crowding index with a high effect size (− 0.87). However, the two groups did not differ significantly in maternal age, parity, gravidity, antenatal depression and anxiety symptoms or infant gestation and birth weight.

**Table 5:** Comparison of significant prenatal differences between invited versus self-referred participants of the feasibility study

Variable	Invited group (*N* = 26) Mean (SD)	Self-referred group (*N* = 74) Mean (SD)	Mean difference (95% CI lower, upper)	Effect size
Education (years)	10.31 (1.78)	9.49 (1.78)	0.82 (0.01, 1.63)	0.46
Monthly income (ZAR)	884 (481)	646 (381)	238 (37, 438)	0.55
Crowding index	1.29 (0.43)	1.92 (0.92)	− 0.63 (− 0.90, − −0.36)	− 0.87
Alcohol (total standard drinks)	2.36 (4.40)	12.04 (23.12)	− 9.69 (− 15.29, − 4.08)	− 0.58
Alcoholic binges (number)	0.08 (0.27)	1.35 (2.64)	− 1.27 (− 1.89, − 0.65)	− 0.68

*N *number, *CI *confidence interval, *ZAR *South African Rands

### Prenatal differences between the feasibility study participants and larger SPS cohort

Next, we compared the demographics and prenatal risk factors between mothers who took part in the feasibility study and mothers of the larger cohort at the time of prenatal enrolment, to ascertain how representative the feasibility sample was of the entire SPS cohort (Table 6). We found nominally significant differences in two variables: During pregnancy, the feasibility study mothers had lived in more overcrowded conditions (mean crowding index, 1.75; 95% CI, 1.58–1.92) compared to the larger SPS cohort [(mean crowding index, 1.57; 95% CI, 1.55–1.59) (effect size, 0.20)] and had scored higher on the Edinburgh Depression Scale [(mean, 14.10; CI, 13.00–15.21) versus (mean, 12.88; CI, 12.74–13.03) effect size 0.21] (NB: a score of 13 or above indicates a high likelihood of depression). There were no significant differences in terms of maternal age, gravidity and parity, education, antenatal anxiety traits, alcohol and smoking during pregnancy, gestational age at delivery and infant birth weight. This indicates that the mothers who participated in the feasibility study were largely representative of the entire cohort.

**Table 6:** Comparison of prenatal characteristics of feasibility cohort (*N* = 100) with safe passage study cohort (*N* = 6874)

Variable	Feasibility study (*N* = 100)	Safe passage study (*N* = 6874)	Comparison
**Maternal variable**	**Mean (SD)**	**Mean (SD)**	***p*-value (uncorrected)**
Maternal age (years)	25.1 (6.2)	24.8 (5.9)	0.780
Gravidity	2.5 (1.5)	2.3 (1.3)	0.163
Parity	1.3 (1.4)	1.1 (1.2)	0.312
Education (years)	9.7 (1.8)	10.0 (1.7)	0.127
Crowding index	1.8 (0.9)	1.6 (0.9)	**0.005**
Household income (Rands)	718 (425)	861 (589)	0.075
Edinburgh depression score	14.1 (5.5)	12.9 (5.9)	**0.043**
Anxiety trait score	41.6 (11.1)	41.0 (10.8)	0.542
Total standard drinks in pregnancy	9.5 (20.4)	13.1 (33.7)	0.274
Binge episodes in pregnancy	1.0 (2.3)	1.3 (3.5)	0.538
Cigarettes per day in pregnancy	2.9 (3.4)	3.0 (3.7)	0.797
Gestational age at delivery (days)	271.0 (16.2)	269.6 (23.0)	0.940
**Child variable**	**Mean (SD)**	**Mean (SD)**	***p*-value**
Birthweight (gram)	3027 (667)	2963 (618)	0.307
Birthweight z-scores	− 0.2 (1.1)	− 0.4 (1.0)	0.194

*SD* standard deviation. Bold *p*-value indicates significant *p* < 0.05

### Feasibility study: clinical and behavioural measures 

Below, we report on comprehensibility and feasibility of all measures, as well as preliminary results of those key measures for which cut-offs are used to inform selection of participants for the deep-phenotyping visit.

#### A. Autistic traits and emotional/behavioural symptoms

##### Social Communication Questionnaire (SCQ)

**Fig. 1:**
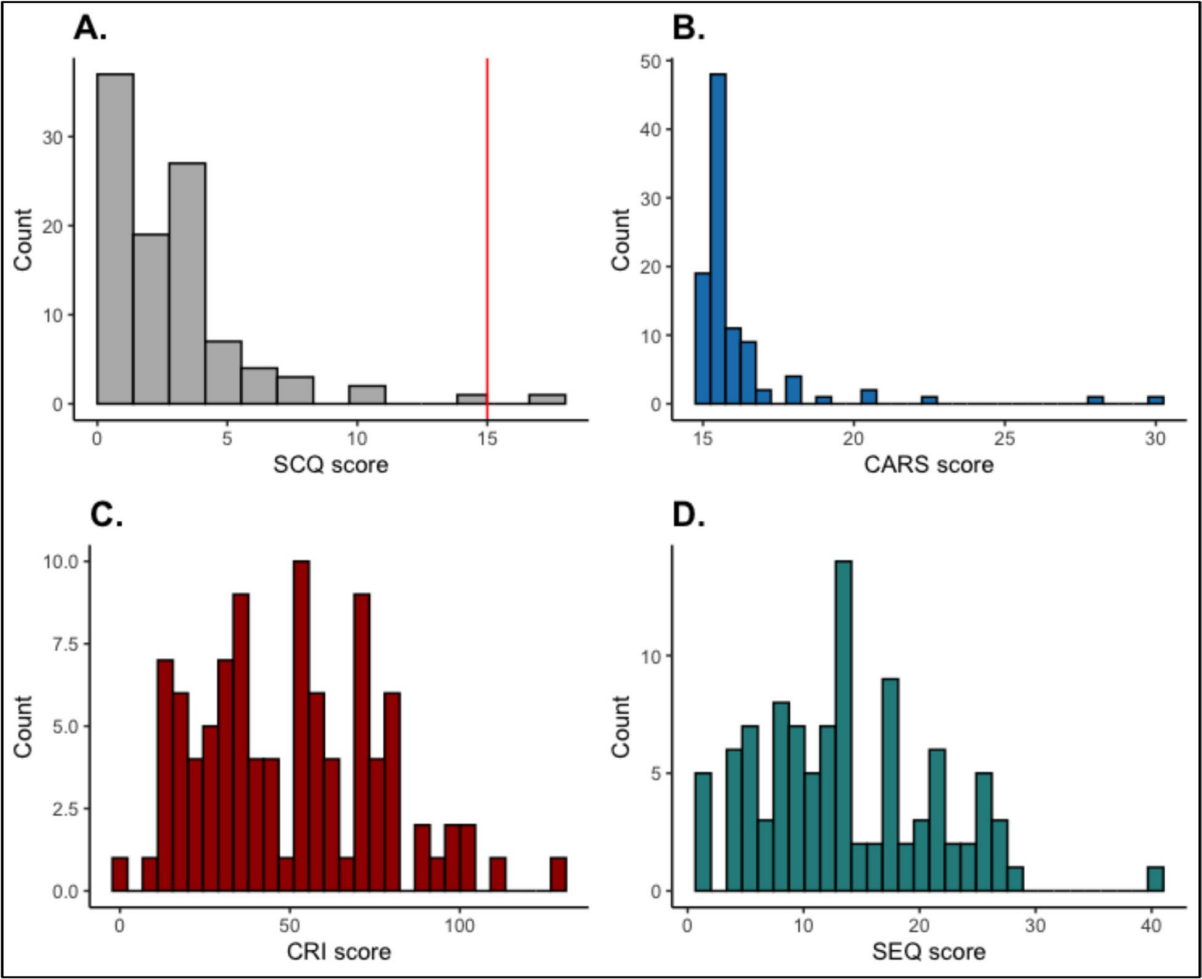
Distribution of scores of autism screening tools and related behavioural measures. **A** Social Communication Questionnaire (SCQ) scores with threshold (cut-off value) of 15. **B** Childhood Autism Rating Scale-revised (CARS). **C** Childhood Routines Inventory (CRI). **D** Sensory Experience Questionnaire (SEQ)

This questionnaire was well understood by most mothers. When reviewing the first 10 participants, testers noted that some mothers initially answered affirmatively to questions about socially inappropriate behaviours (question 4), compulsive routines (question 8) and unusual sensory interests (question 14), but when asked to give examples, they rescinded the positive response. Thus, research workers asked caregivers to elaborate and gave explanations and examples of autism-related behaviours, reassuring them that a negative response was expected for most questions. Figure 1 shows the distribution of raw SCQ scores. As expected for the use of an autism screening measure in a community cohort, the distributions were skewed towards subthreshold scores. The manual recommended a cut-off score of 15. Two children (participants 4 and 5) scored at or above this Western cut-off both of whom subsequently had autism classification confirmed on the Autism Diagnostic Observation Schedule-second edition (ADOS) and CARS (Appendix 4). There were no significant sex differences in SCQ total score (*t* = − 1.07, df = 99, *p* = 0.28). The number of autistic features did not correlate with IQ (*r* = − 0.01, *p* = 0.93) and was negatively related to age (*r* = − 0.26, *p* = 0.01) such that younger children tended to have more autistic traits.

##### Childhood Autism Rating Scale-revised (CARS)

The research workers reported no major problems with the measure, which was well understood by the caregivers. As expected, CARS scores were skewed towards scores below the clinical range. The frequency of children who scored around cut-off on the SCQ and CARS was broadly in keeping with expectations from Western HIC studies, which currently estimate that approximately 1 in 58 individuals are autistic [57]. The high correlation between SCQ and CARS-total scores (*r* = 0.75, *p* < 0.0001) was reassuring as both children who scored above the SCQ threshold did so on the CARS. In contrast to the SCQ, CARS total scores were strongly negatively correlated with Full-Scale Intellectual Quotient (FSIQ) (*r* = − 0.71, *p* < 0.0001) but not with age (*r* = − 0.04, *p* = 0.75). Again, there were no statistically significant sex differences (t(97) = − 0.67, *p* = 0.58).

According to the original protocol, we planned to use the CARS to confirm an autism diagnosis during deep phenotyping, and we found that both children scoring above the threshold on the SCQ received autism classification on the CARS. However, several AIMS-2 experts raised concerns about the use of the CARS as a diagnostic versus screening instrument (Prof. Ed Cooke, Prof. Emily Simonoff). The recent translation of the ADOS into Afrikaans enabled us to use this “gold standard” measure instead [58]. The two children with high SCQ scores were subsequently assessed on the ADOS, and both exceeded threshold for ASD. We then took an additional two children from the Tygerberg Hospital Developmental Clinic who were not in the feasibility study but had a confirmed autism diagnosis and assessed them on the CARS and ADOS. The ADOS correctly classified both children with autism, whereas the CARS missed the diagnosis in one of them. Based on this combined information, we decided to replace the CARS with the ADOS, as it was more sensitive (Appendix 4) in the understanding that it is frequently used alongside the autism diagnostic interview, which could not be implemented due to time constraints.

##### Abridged Childhood Routines Inventory (CRI-R)

Feedback from testers and participants was that items were well understood, although some participants found it too long. Scores were normally distributed in line with the intention of the scale to be used in community samples. In the current analyses, raw scores were used, as the scale has not been validated in South Africa; thus, we could not compare with US norms. CRI total scores were negatively correlated with full-scale IQ (*r* = − 0.28, *p* < 0.01) but positively with age (*r* = 0.017, *p* = 0.11). There were no sex differences (*t* = − 0.38, df = 96, *p* = 0.69).

##### Sensory Experience Questionnaire (SEQ)

**Fig. 2:**
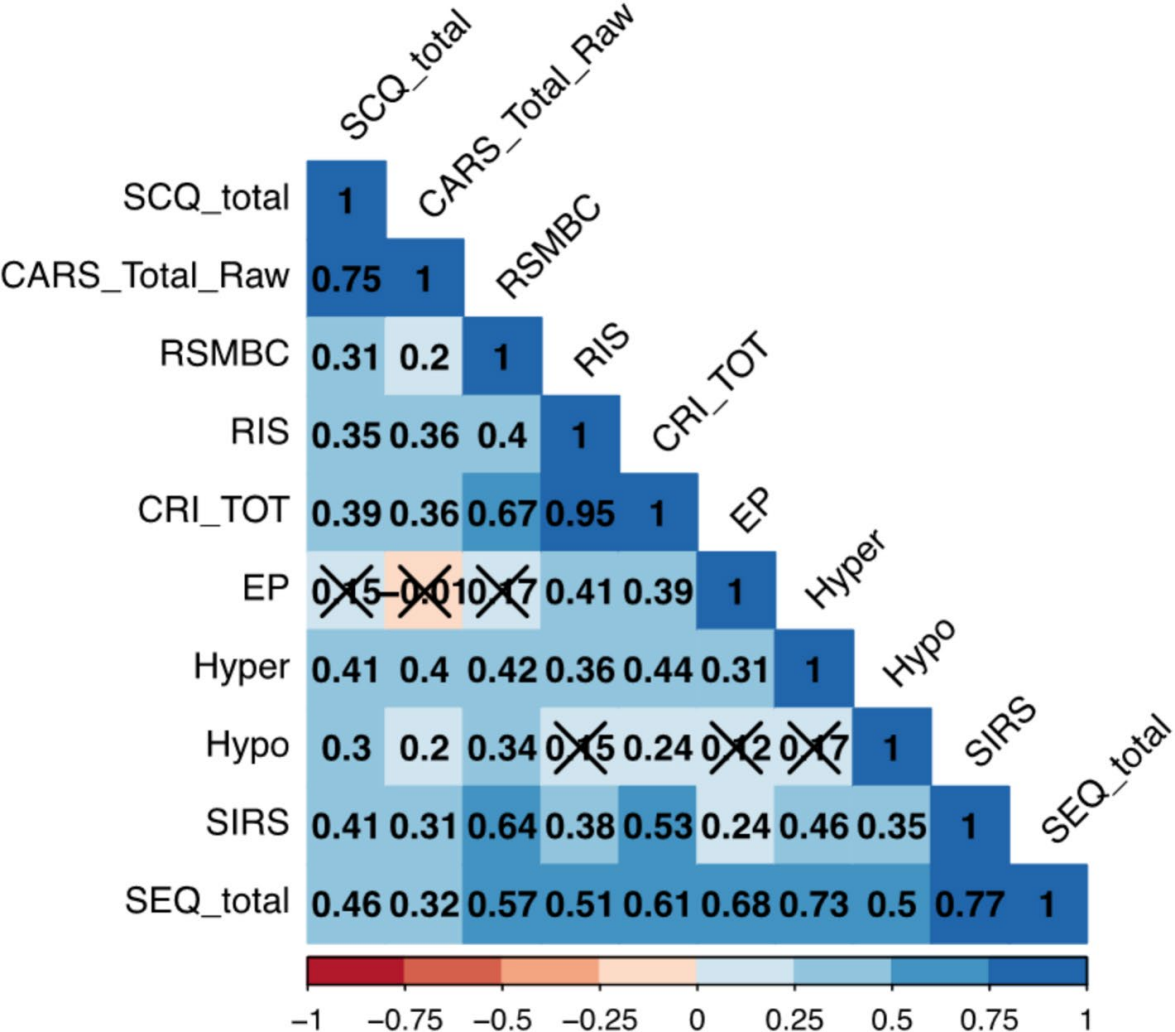
Correlation between autism core features. Key: CARS_Total_Raw, Childhood Autism Rating Scale-revised, Total Raw score; SCQ, Social Communication Questionnaire total score; CRI-R_TOT, Childhood Routines Inventory-Revised Total score; RIS, Rigidity and Insistence on Sameness subscale; RSRMBC, restricted sensory and repetitive motor behaviours and compulsions subscale; EP, enhanced perception; Hyper, hypersensitivity; Hypo, hyposensitivity; SIRS, sensory interests, repetitions and seeking behaviours; SEQ_total, Sensory Experience Questionnaire total score; correlations marked by “X” were non-significant at *p* = 0.05

Feedback from the testers was that this questionnaire was clearly understood by most mothers and the items provided sufficient examples. Scores were broadly normally distributed. SEQ scores were not significantly related to FSIQ (*r* = − 0.12, *p* = 0.3). There were no relationships with age (*r* = 0.09, *p* = 0.38), and again no sex differences (*t* = − 0.37, *p* = 0.7). As shown in Fig. 2, there were moderately strong correlations between the SCQ and repetitive behaviours (CRI-tot, *r* = 0.39, *p* < 0.001) and sensory features (SEQ total, *r* = 0.46, *p* < 0.001). Furthermore, in line with expectations, repetitive behaviours and sensory features were highly correlated (rs > 0.6).

##### Strengths and Difficulties Questionnaire (SDQ)

**Fig. 3:**
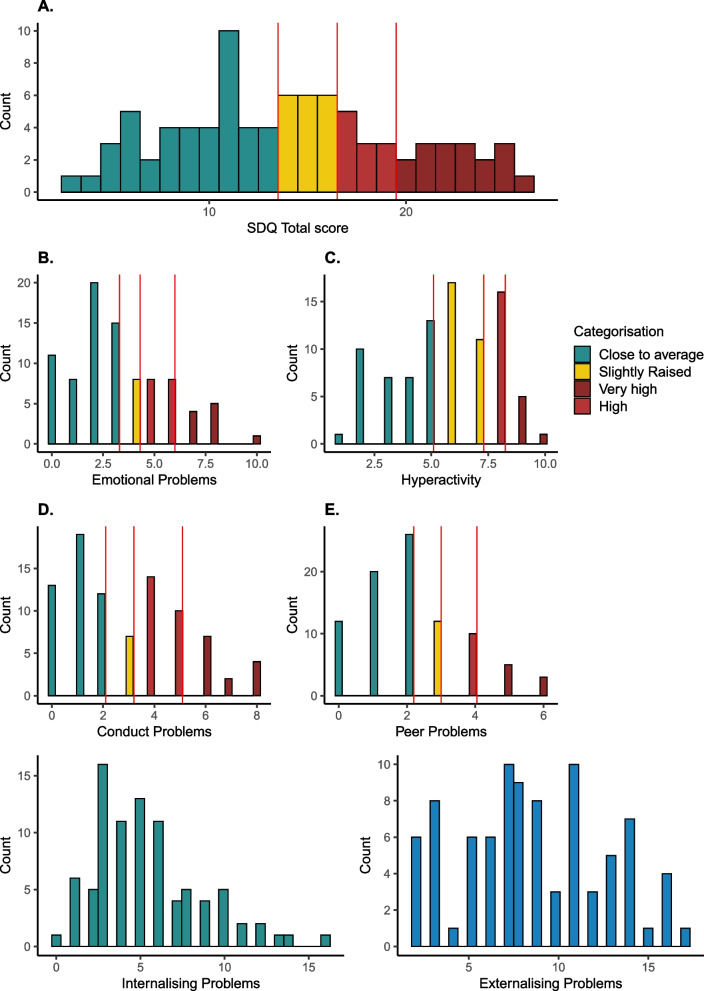
Distribution of Strengths and Difficulties Questionnaire scores. Total **A** and subscale scores, **B** emotional, **C** hyperactivity, **D** conduct and **E** peer problems. Combined internalising (**B** + **E**) and externalising scores (**C** + **D**)

The overall response from testers and participants to this measure was positive. The questions were clear and easily understood. Some caregivers (6%) elected to complete this questionnaire without assistance from the research team members. Only 87 of the 100 children aged 4 − 12 years were included in the present analyses as this more accurately reflected the age of the main cohort going forward. The mean total difficulties score was 13.5 (upper limit of average range), SD 6.1 and range 1 − 28. There were 12.5% of children with “high” and 19.3% “very high” total difficulties scores, 20.4% had “slightly raised” scores and 47.7% scores “close to average” (Fig. 3A). In comparison, UK norms anticipate 5% for both “high” and “very high” difficulties [59]. Impact scores were collected later in the study (63 participants), but only 5 caregivers of children with “high” or “very high” scores reported significant impact of the child’s behaviour on family functioning.

The data of 13 children younger than 4 years were analysed separately, and 6 (46%) had a total difficulty score in the high or very high range, with 3 caregivers (23%) reporting impact scores of 1 or higher. However, these “cut-off” scores are provisional due to lack of a large nationally representative sample for standardisation.

The frequency of emotional, social, conduct and hyperkinetic symptoms was also significantly elevated compared to Western reference scores [44]. When split by subscales (Fig. 3B and C), the highest scores were found in terms of externalising behaviours. Compared to SDQ Western norms, only 50% of children scored “close to average” in terms of conduct problems; 8% had slightly raised scores, 27.2% high and 14.7% very high scores. Likewise, 43.1% had “close to average” hyperkinetic symptoms, while 31% had “slightly raised”, 18.1% “high” and 8.6% “very high” scores.

Internalising scores were also high. For emotional symptoms, 9.1% scored in the “slightly raised range”, 18.1% “high” and 11.3% “very high”. For peer problems, 13.6% had “mildly raised”, 11.3% “high” and 9% “very high” scores. IQ was significantly related to emotional symptoms (*r* = − 0.33, *p* = 0.007), marginally significantly to conduct problems (*r* = − 0.22, *p* = 0.07) but not significantly related to peer problems (*r* = − 0.17, = 0.17) or hyperactivity (= − 0.09, = 0.45). Age was not related to either internalising or externalising scores (hyperactivity: *r* = − 0.04, *p* = 0.70; conduct: *r* = − 0.1, *p* = 0.34; peer: *r* = 0.05, *p* = 0.64; emotion: *r* = 0.09, *p* = 0.38). In contrast to numerous previous reports of sex differences in internalising/externalising behaviours, boys and girls did not significantly differ in terms of their SDQ total scores or emotional, peer, hyperkinetic or conduct problems except for a nonsignificant trend for more externalizing behaviours in boys (*t* = 1.81, df = 92, *p*-value = 0.07). There were also no sex differences with regard to prosocial behaviour (*t* = − 0.71, df = 92, *p*-value = 0.48).

##### Childhood Oxford-Liverpool Inventory of Feelings and Experiences (CO-LIFE)

The CO-LIFE is a dimensional parent-report measure of schizotypal and psychotic traits intended for use in the general population to detect prodromal features of schizophrenia across childhood. Some participants found certain CO-LIFE questions to be strange and easily misinterpreted; Questions 3 (child feeling easily overwhelmed), 5 (extreme mood swings), 6 (thought rumination) and 13 (unable to let bad thoughts or experiences go) needed clarification. The testers addressed this by explaining how these questions were related to child mental health, provided examples and encouraged discussion and questions which led to them rescinding some of the positive responses. The distribution was strongly skewed towards no or few (< 5) schizotypal symptoms, with 6 children being reported to display 10 or more symptoms. This is in line with previous reports, given the nature of some items probing for hallucination and delusion. Schizotypy scores were significantly negatively related to IQ (*r* = − 0.41, *p* < 0.0001) but not age (*r* = 0.02, *p* = 0.84), and there were no sex differences (*t* = − 0.75, df = 11, *p* = 0.45).

##### The Child Behaviour Questionnaire (CBQ)

**Fig. 4:**
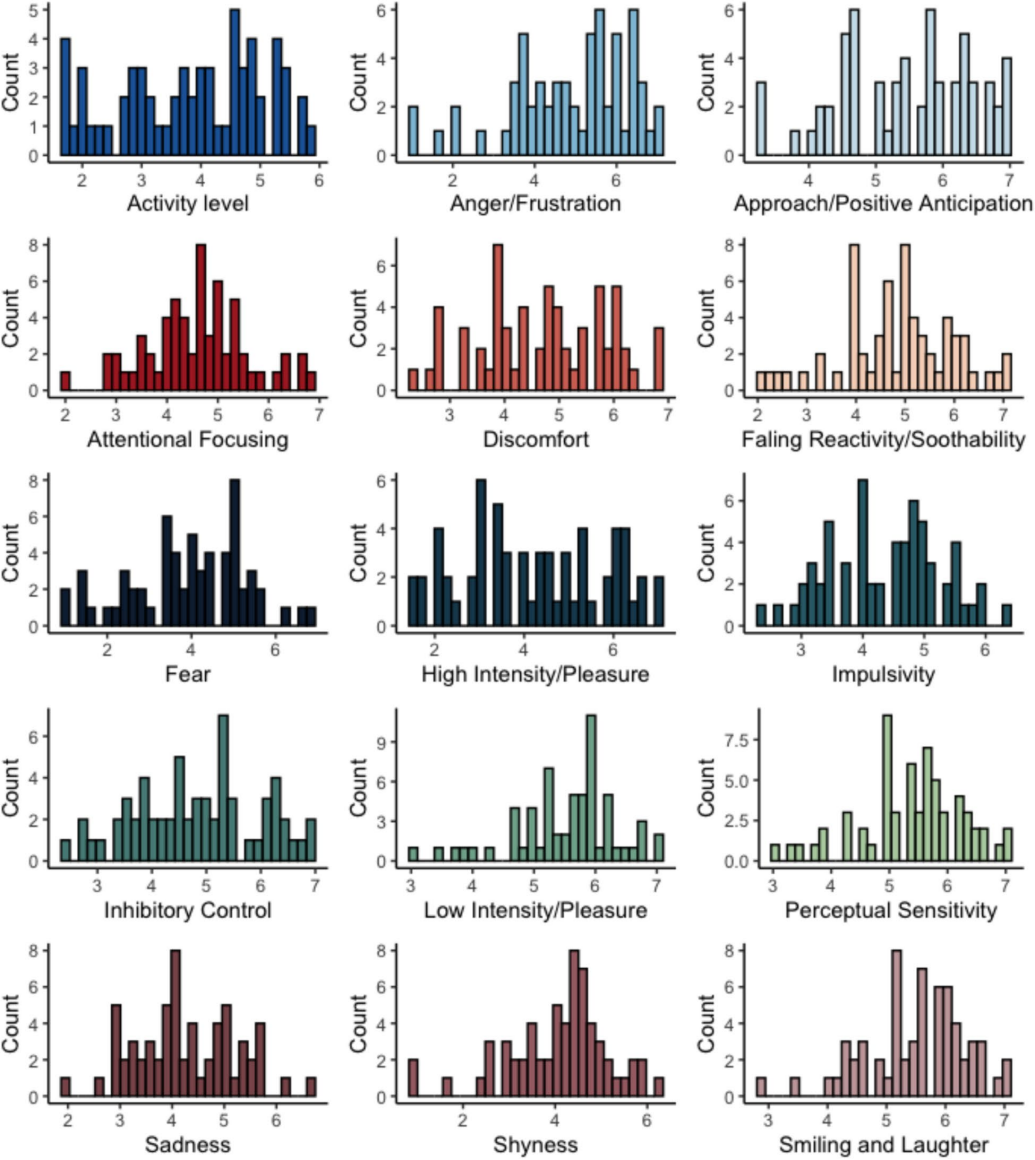
Distribution of child behaviour questionnaire subdomain scores (*N* = 62)

This questionnaire was reported to cause participant fatigue. It was evident that several questions resembled those asked in the other mental health questionnaires. A total of 62 questionnaires were administered before discontinuation. Distribution of scores, by subdomain, are shown in Fig. 4.

#### B. Maternal autism and mental health measures

**Fig. 5:**
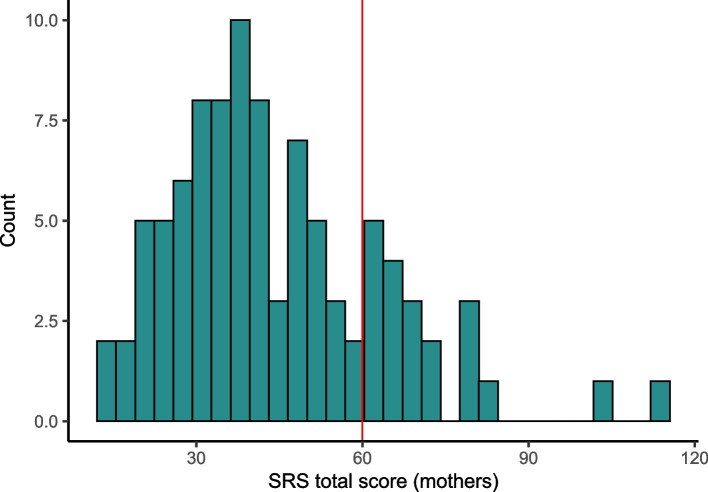
Distribution of social responsive scale questionnaire scores of the mothers

Research workers reported that the SRS-2 questionnaire (65 items), which screened for autistic symptoms in the caregiver, was found to be burdensome by the majority of the participants, and the questions were difficult to understand. Distribution of mothers’ scores is shown in Fig. 5, and 20 out of 94 mothers (21%) scored within the autism range, i.e. total score was above 60 points.

Acquisition rates of questionnaires were high (Table 7). The maternal self-reported SDQ symptom scores (*n* = 95) were elevated with a mean total of 16.9 (SD, 5.7; range, 7–34) which was higher than that of the children (mean, 14.1; SD, 6.8; range 1–31). Of the 26 mothers (27%) with very high total scores ≥ 20 (mean, 24.8), many had documented prenatal risk factors, in particular high anxiety trait scores (40%), Edinburgh score > 18 (58%), crowding index > 2 (42%), less education < 9 years, parents not living together (42%), heavy smoking > 6.5 cigarettes per day (27%), previous miscarriage, stillbirth or infant loss (23%), recreational drug use (19%), “no phone” as a proxy for poverty (19%), high gravidity > 5 pregnancies (8%) and high alcohol intake (4%). Children of these mothers had a mean total score of 18.4 (high range), and 14 (54%) children had total scores ≥ 20 (very high range).

**Table 7:** Acquisition, implementation, adaptation and final decision on measures trialled in the feasibility study including real times

Measure (estimated time)	Total no. of tests	No. of timed tests	Real time min (SD)	Range (min)	Adaptations	Final decision
Demographic form (15 min)	100	46	5 0 (2.5)	3–12	None	Included
RPQ (10 min)	38	38	15.4 (7.2)	7–43	ACES added	Included
MSEL (15 min)	35	20	48.0 (11.6)	30–75	Appendix 2	Included
WASI child (20 min)	64	36	34.0 (9.0)	19–50	Appendix 2	Included
WASI mother (40 min)	83	37	41.9 (10.8)	22–64	None	Included
SCQ (15 min)	100	94	9.8 (5.4)	3–37	Examples and clarification	Included
SDQ child (7 min)	100	92	7.3 (4.2)	1–28	None	Included
SDQ adult (7 min)	94	92	5.7 (3.2)	2–19	None	Included
SEQ (7 min)	98	95	7.4 (3.2)	2–21	None	Included
CBQ (25 min)	62	60	23.3 (6.3)	14–45	None	Excluded
CRI-R (11 min)	96	87	15.8 (5.2)	4–26	None	Included
CO-LIFE (4 min)	100	95	4.0 (2.8)	2–15	None	Included
O-LIFE (4 min)	94	91	4.0 (2.6)	1–23	None	Included
SRS-2 (15 min)	93	92	16.3 (6.3)	7–35	None	Excluded
CARS-QPS (30 min)	94	91	14.4 (6.4)	6–37	Compared with ADOS	ADOS substituted
EEG capping (10 min)	130	103	16.7 (5.1)	7–39	None	Included
EEG recording (20 min)	130	104	22.8 (2.3)	17–37	Technical adjustment	Included

Key: *ACE* adverse childhood experiences, *ADOS* Autism Diagnostic Observation Schedule-second edition, *CARS* Childhood Autism Rating Scale-revised, *CBQ* Child Behaviour Questionnaire, *CO-LIFE* Childhood Oxford-Liverpool Inventory of Feelings and Experiences, *CRI-R* Childhood Routines Inventory-Revised, *EEG* electroencephalogram, *MSEL* Mullen Scales of Early Learning, *O-LIFE* Oxford-Liverpool Inventory of Feelings and Experiences, *RPQ* Risks and Protective factors Questionnaire, *SCQ* Social Communication Questionnaire, *SDQ* Strengths and Difficulties Questionnaire, *SEQ* Sensory Experience Questionnaire, *SRS-*2 Social Responsiveness Scale, *WASI* Wechsler Abbreviated Scales of Intelligence-second edition

Questions in the O-LIFE (mother’s self-report about herself) elicited emotional responses in a few participants and highlighted some adult participants expressing intention to self-harm (suicide attempts). The team referred these participants with their permission to the appropriate mental health services. Religious, spiritual and cultural backgrounds may have influenced the interpretation of some questions. As in the children, mothers’ schizotypy scores were skewed towards no or few symptoms and were moderately correlated with IQ (*r* = − 0.41, *p* < 0.0001), but not with age (*r* = 0.12, *p* = 0.24).

Mother–child intra-class correlations (ICC = 0.22, *p* = 0.02) were significantly lower than those reported from a large US sample, which reported high ICCs (0.80) [46]. This is likely due to the small sample size and consequently small proportion of scores in the clinically relevant range.

#### C. Cognitive/developmental measures

When designing the protocol, the Leiter-III was selected as a non-verbal intelligence and cognitive test, due to its supposedly culture-fair administration mode. However, initial discussions amongst the team (around January 2019) revealed that the non-verbal format seemed to confuse the youngest children. Hence, we used the Mullen Scales of Early Learning (MSEL) for children up to age 6 years and the WASI for children from the age of 6 years.

##### Mullen Scales of Early Learning (MSEL)

**Fig. 6:**
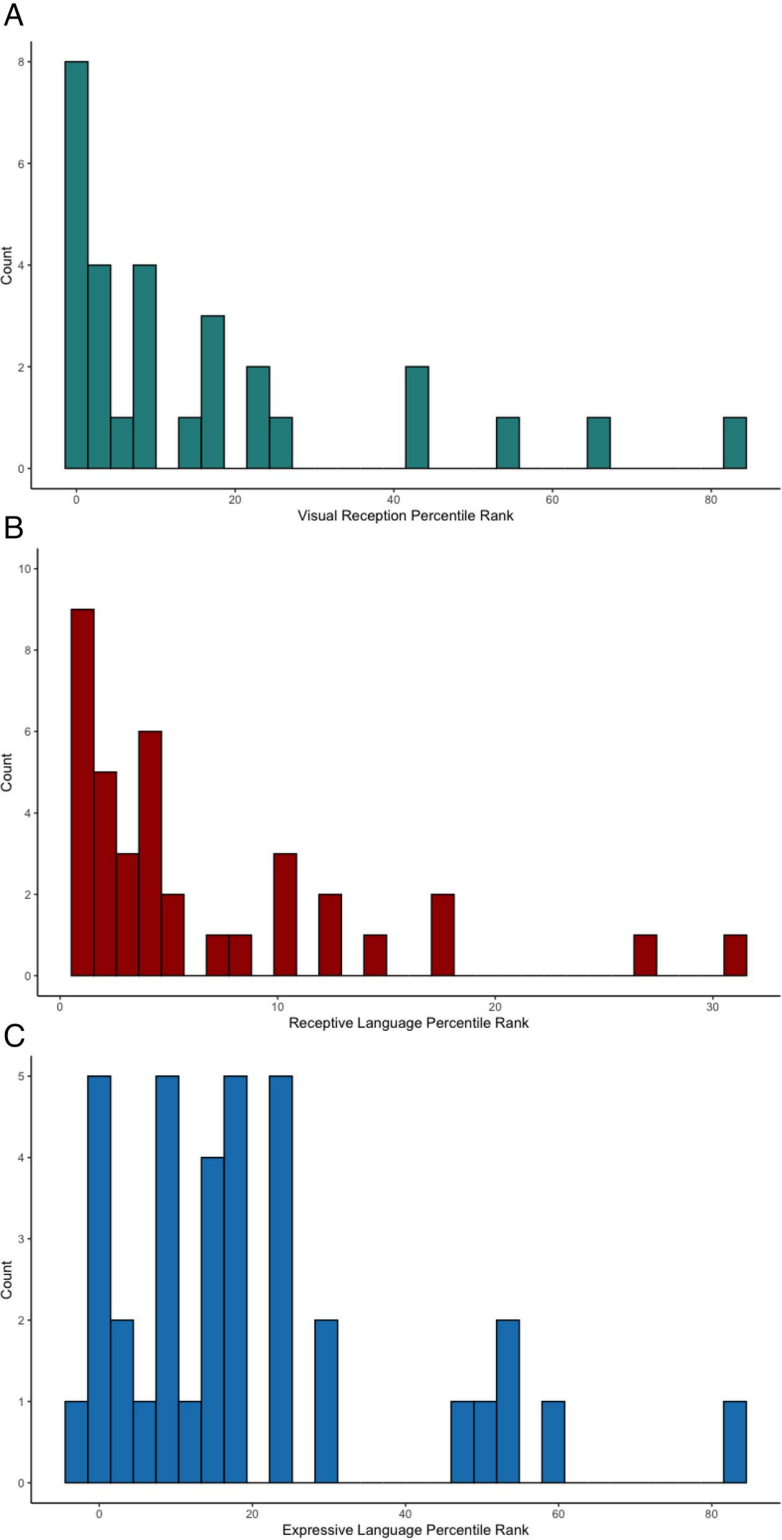
Domain percentile ranks of Mullen Scales of Early Development: **A** Visual Reception **B** Mullen Scales of Early Development: Receptive Language **C** Mullen Scales of Early Development: Expressive Language

The three MSEL subscales—visual reception, receptive language and expressive language—were strongly skewed towards low performance. On the visual reception subscale (Fig. 6A), 11 children (37%) performed within the average range for their age, eight (27%) below average and 10 (34%) very low (1st percentile) (US norms). In terms of receptive language (Fig. 6B), only 4 children (9%) scored within the average range for their age, 19 (46%) below average and 14 (34%) very low. On the expressive language subscale (Fig. 6C), 18 (51%) children performed within the average range, 11 (31%) below average and 6 (17%) very low.

##### Wechsler Abbreviated Scales of Intelligence-second edition (WASI*)*

The instructions and items were overall well understood. On the vocabulary subscales, a few adaptations were made for words that were culturally unfamiliar, varied in colloquial meaning or lacked comparable Afrikaans equivalents (Appendix 2). In some cases, the Afrikaans word was more descriptive and self-explanatory than the English, lacking equivalence in degree of difficulty. Team consensus was obtained before making substitutions or adaptations. Code switching, i.e. changing language throughout a conversation, was common, and in some instances, the English-speaking children were more familiar with the Afrikaans translation than the English equivalent, e.g. “haastig” versus “haste”.

**Fig. 7:**
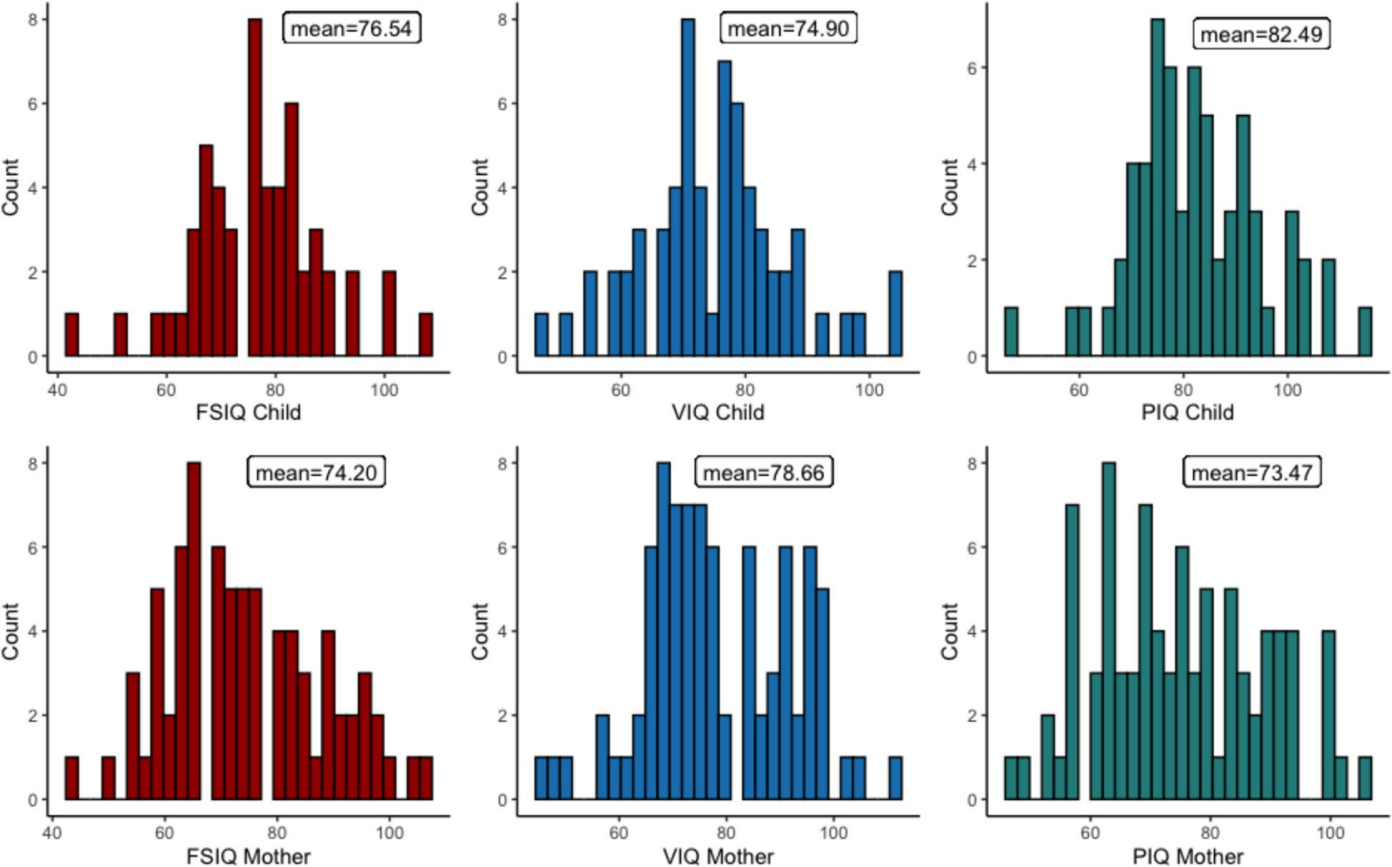
Distribution of WASI FSIQ, VIQ and PIQ: upper panel, child; lower panel, mother. Key: WASI, Wechsler Abbreviated Scales of Intelligence, second edition; FSIQ, Full-Scale Intelligence Quotient; VIQ, verbal intelligence quotient; PIQ, performance intelligence quotient

**Fig. 8:**
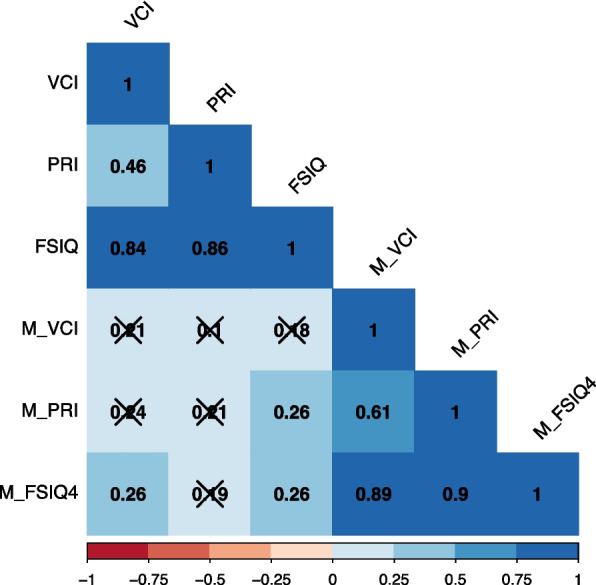
Heat map showing correlations between mother and child IQ scores

In contrast to the MSEL, children’s and mothers’ data on the verbal and performances subscales of the WASI, and the full-scale estimate (FSIQ), were normally distributed. However, in children, VIQ and FSIQ scores were shifted about 25 points and 17 points, respectively, below standardised Western HIC means and distributions. A similar shift has been found in norming studies of the Wechsler Intelligence Scale for Children, Fourth Edition, in South African adults and children by Shuttleworth (2016) who related performance to level and quality of education, emphasising that standardisation was required for multicultural settings [60, 61]. Because of the shift in the score distribution, a cut-off for “low WASI” was defined as VIQ, PIQ and/or FSIQ scores (any scale) of 60 or below for referral for deep phenotyping. Despite relatively similar distributions and performance means, correlations between mother and child IQ were low to medium (rs ≥ 21 ≤ 0.26, see Figs. 7, and 8).

##### Risk and protective factors questionnaire (RPQ)

To assess adverse childhood experiences (ACEs), a parent (caregiver)-report Risk and Protective Questionnaire (RPQ) was adapted from existing instruments. This included all items comprising the Adverse Childhood Experiences-International Questionnaire (ACE-IQ) (WHO, 2018) [47], which was designed to measure ACEs in all countries. To this, we added selected items from the Childhood Trauma Questionnaire[62] and the IPSCAN Child Abuse Screening Tool-Parent version (ICAST-P) [63]. Together, they assess abuse/neglect, family dysfunction, family hardship and community violence. We also probed for protective factors pertaining to social relationships between mother–child, family, other significant adults (e.g. teachers) and peers and to individual characteristics. This included selective items from the Connor-Davidson Resilience Scale (CD-RISC2) [64], as well as a section on children’s daily routines, including questions on nature and frequency of play, story telling/reading books, TV, etc. In addition to the original response format of the ACE-11, which scores whether or not an event occurred, we probed for frequency, who (in the family) was involved and impact of the event on the child (through the lens of the parent-informant). The measure was introduced after the feasibility study had started, and only 38 participants completed the questionnaire. Participants found it comprehensible and relevant. The median number of ACEs was 5. Ten children (26%) had even experienced eight or more risk factors. Although the questionnaire used in the feasibility study was not the same as the ACE-11, it is of note that the Center for Disease Control (USA) considered two ACEs to be a risk factor and four or more ACEs as putting a child at very high risk for mental disorders. In the US sample, 4 + ACEs were only found in approximately 15% of participants. This indicates a very high rate of ACEs in the present sample.

##### EEG and eye-tracking results

**Fig. 9:**
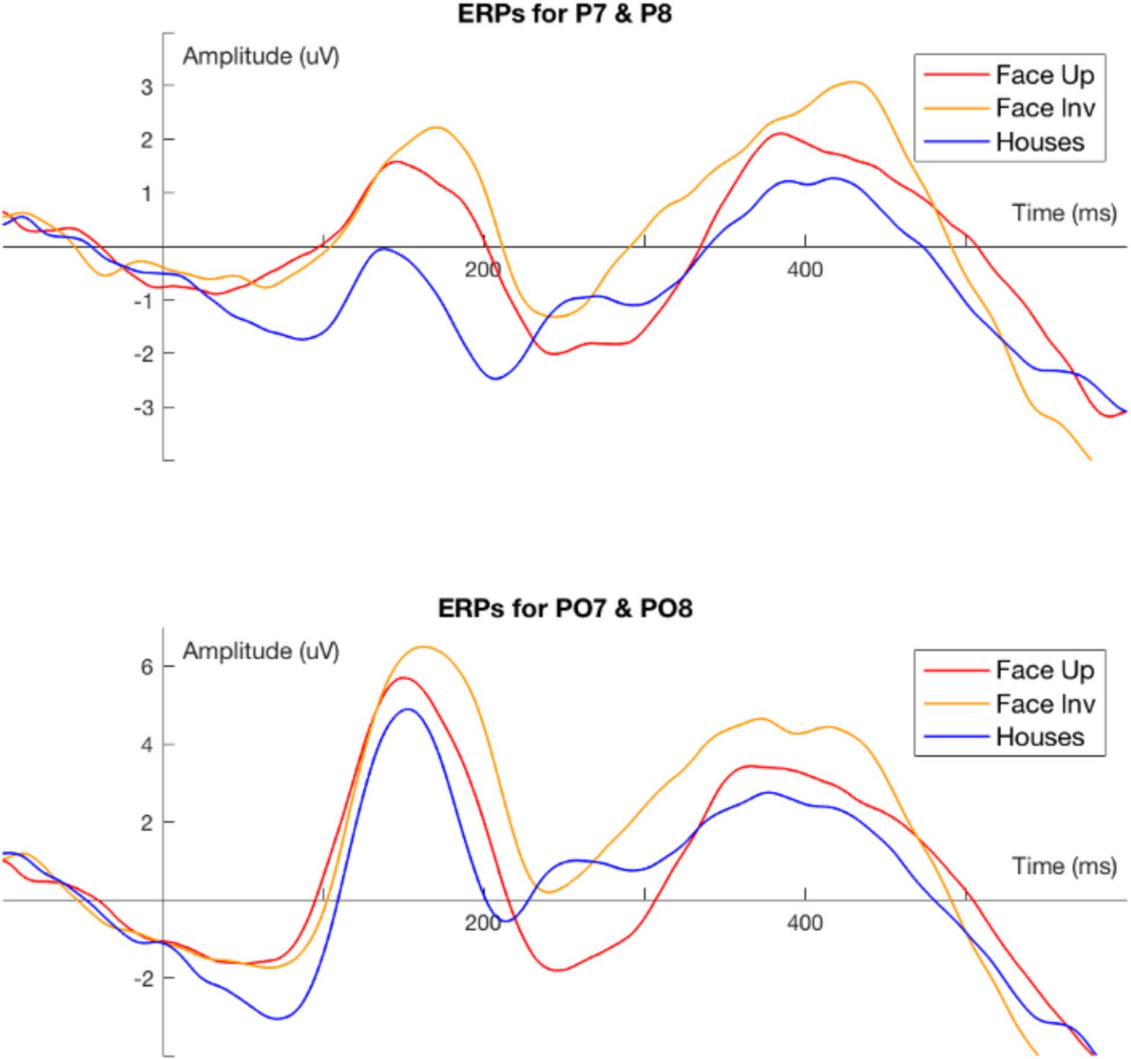
Grand average of event-related potential task (ERP) examining the face inversion effect from the interim analysis (25 participants)

All 100 children agreed to participate in the EEG and eye-tracking procedures (100% acquisition). Throughout the study, interim quality checks on 25 randomly selected participants revealed that the average 50-Hz noise decreased across the months of testing, and the number of presented trials did not vary with age, suggesting good compliance with the procedure. Furthermore, the grand average of the event-related potential from the faces task revealed a visible P1 component and N170/290 components for upright and inverted faces (Fig. 9). All 25 participants contributed at least 20 out of 72 trials per condition, indicating optimal feasibility for the dynamic videos. Seventy-four of the 100 participants provided EEG and eye-tracking data with the women singing nursery rhymes and spinning toys. Among them, 72 exhibited good signal quality, artefact-free epochs and an average percentage looking time > 80%. Aperiodic-adjusted components showed significantly different spontaneous patterns during the social and nonsocial resting-state conditions, indicating promising sensitivity to brain responses for use in the larger sample [50].

##### Touch screen tests

Early beta versions of touch screen tests assessing inhibitory control (Go-NoGo “Puppy task”), sustained attention (“Pip’s car”), reward learning (“Magic boxes”), attentional biases to happy/fearful faces (emotional dot probe “Catch the Butterfly”) and two theory of mind tests (false belief “Pip’s bus” and false belief word learning “What’s a modi?”) were administered to 22 children. Due to the small sample, data (including children’s comprehension of task instructions, trial number, surface design features and performance) were analysed qualitatively and informed further task development. Subsequent task versions of a companion test battery “Time Trekkers” for children aged 6 years and older were included in broad and deep-phenotyping phases of the main protocol.

### Acquisition rates and overall testing time

There was 100% acquisition rate for all questionnaires and cognitive tests, although some measures were discontinued or introduced later during the feasibility study. Eighty participants were timed. The consenting procedure took on average 13.7 min with a maximum of 26 min (range 5–26 min). Total time of the broad phenotype visits varied between 120 and 140 min and the deep phenotype between 120 and 150 min which was within the anticipated limits and allowed for comfort breaks.

### Clinical referrals

Twenty-one children required referral for further diagnostic evaluation of detected conditions, including audiology and speech therapy (7), developmental assessment clinic (5), mental health services (3), occupational therapy (2), ophthalmology clinic (1) and paediatric outpatients (3).

### Final protocols

The final protocol for the broad phenotyping is shown in Table 8, while the final protocol for the deep phenotyping is shown in Table 9. These measures will be implemented in the main Safe Passage BONO study.

**Table 8:** Final revised protocol of caregiver and child measures for broad phenotyping (Visit 1)

	Domain	Measure
	Child health information	Weight, height, head circumference, blood pressure
Parent (about child)	Past medical/family history	Demographics and medical history questionnaire
	Autistic traits	Social Communication Questionnaire (SCQ)
	Mental health	Strengths and Difficulties Questionnaires (SDQ) Childhood Oxford-Liverpool Inventory of Feelings and Experiences (CO-LIFE), schizotypy subscale
	Adaptive function	Vinelands Adaptive Behaviour Scales-3rd edition
	Childhood adversities and protective factors	Risk and Protective factor Questionnaire 24 items (stressful life events and protective support)
	Total time(parent):	**Estimated time for parents: 80 min** (plus breaks)
Child	Language development/verbal/non-verbal/cognition	Age 3–6: Expressive/receptive subtests of the Mullen Scales of Early Learning (MSEL) Age 6–12 (or in younger child if ceiling on MSEL is reached): Wechsler Abbreviated Scales of Intelligence-second edition (WASI) vocabulary/similarity subscales/matrix reasoning/block design
	Tablet tasks	Tasks evaluating inhibitory control, sustained attention, reward learning, emotion recognition, theory of mind
	EEG tasks*	Event-related potential task, three resting-state conditions, measurement sensory processing efficiency
	Total time (child)	**Estimated time for child: 85 min** (plus breaks)

**Table 9:** Final revised protocol of caregiver and child measures for deep phenotyping (Visit 2)

	Domain	Measure
Parent	Autism assessment for children with SCQ ≥ 10	Autism Diagnostic Observation Schedule-2
	Repetitive behaviours and restricted interests in child	Childhood Routines Inventory-Revised (CRI-R)
	Sensory atypicalities in child:	Sensory Experience Questionnaire (SEQ)
	Child mental health DAWBA (for children with SDQ scores, i.e. total difficulties score or any subdomain) in “very high” range or WASI Full-Scale Intellectual Quotient < 60	Developmental and Well-Being Assessment (child) (DAWBA)
	Adult mental health symptoms	Strengths and Difficulties Questionnaire (adult self-report version) (SDQ) Oxford-Liverpool Inventory of Feelings and Experiences O-LIFE (schizotypy subscale)
	Maternal cognition	Wechsler Abbreviated Scales of Intelligence-second edition (WASI)
	Total time (parent)	**Estimated time for parent: 135 min** (plus breaks)
Child	Tablet tasks	Tasks assessing decision-making under uncertainty, preference for social novelty and sensory processing (frequency discrimination)
	Total time (child)	**Estimated time for child: 70 min** (plus breaks)

## Discussion

We have outlined the design and measures to be used in the Safe Passage-BONO study and presented the results of a feasibility study with 100 mother–child dyads. Key criteria [38] for estimating feasibility and likely study success included the following: (1) Recruitment and retention capability; (2) suitability of measures and procedures in the present cohort; (3) feasibility and burden of the protocol for testers and families; (4) determination of cut-off scores, i.e. eligibility for deep phenotyping; (5) adequate resources to manage and implement the study; and (6) appropriate referral pathways for the current study population.

### Recruitment/retention capability

Adequate recruitment of SPS study participants was evidenced in our study similar to that experienced in the previous follow-up studies involving SPS pre-schoolers [31–33]. Approximately, 50% of participants responded to initial hand-delivered letters within 2 days; however, recruitment was substantially increased and accelerated by “word of mouth”. There were significant differences between those participants who responded to invitation letters and those who independently contacted the research unit. It is possible that parents contacting the unit had more pressing financial needs or concerns regarding their children.

Comparison of demographics and prenatal risk factors between the feasibility study sample (100 participants) and the original SPS cohort also showed differences in key demographic and prenatal risk variables at the time of original enrolment. Thus, the feasibility study participants were partly a sample of convenience rather than representing random selection from the entire cohort.

### Suitability/acceptability of outcome measures and experimental tasks

It became evident that research workers, with institutional and local knowledge, had previously forged positive relationships with participants during the SPS. They were familiar with the Kaaps dialect, which is unique to the Western Cape [51], and were well-placed to assist in selection, translation, evaluation and adaptation of standardised measuring tools.

Three measures were used in the broad phenotype visit specifically for selection of children for deep phenotyping: the SCQ (autistic traits), SDQ (internalising and externalising scores) and WASI or MSEL (intellectual function), and all were included in the final BONO protocol.

The SCQ did not require adaptation. The testers minimised potential over-reporting by explaining to caregivers that they anticipated a negative response for most questions and asking for examples of reported behaviours. Selection of the SCQ is supported by a recent systematic review of autism screening tools used in Africa, which recommended the SCQ as appropriate for detection of autism in children and adolescents [65]. However, like most screening tools (except for the M-CHAT that is only suitable for toddlers), it has not been validated in South Africa [66]. Research workers were later enrolled in the AIMS-2-TRIALS Autism Diagnostic Interview-Revised (ADI-R) training to increase their awareness of autism-related behaviours. As indicated above, despite good feasibility results, we elected to replace the CARS with the ADOS.

The selection of cognitive measures proved more challenging. Before the start of the feasibility study, six children were tested on the Leiter-III scales, a nonverbally administered measure of fluid IQ, which was considered more “culture fair”. However, when research workers reported that notably the younger children were confused by this non-verbal administration procedure, the Leiter-III was replaced by the MSEL in children < 6 years and the WASI in children from 6 years onwards. Using these tests also enables us to compare findings on the WASI between child and parent and to compare the Safe Passage BONO cohort with other EU-AIMS/AIMS-2-TRIALS clinical research studies (LEAP, SynaG, PIP) [67, 68]. The bilingual nature of the cohort meant that participants often switched between languages in the same conversation, and for this reason, it was decided that participants would be provided with both English and Afrikaans versions. The verbal subscales of the WASI required minor adaptations to include more culturally familiar words and pictures as did the MSEL (Appendix 2).

### Burden of protocol on testers and families

The concurrent evaluation of mother and child allowed for efficient use of time and reduced participant fatigue or boredom. Acquisition rates, compliance and participant satisfaction were high for most questionnaires and experimental measures.

Clinical and behavioural questionnaires were read to caregivers by the research workers to ensure understanding and allow opportunities for questions. It was found that this administration procedure was acceptable to participants and could be completed within the time frame. It also enabled the researchers to add examples in instances where mother had difficulties understanding specific questions/items. The high tolerance of EEG procedures in this cohort was notable.

### Preliminary analyses and decisions on cut-offs for “deep phenotyping”

Preliminary analysis showed that after providing participants with the additional explanation and examples early on in the feasibility study, the frequency of autistic traits in this sample was broadly similar to expectations based on global prevalence rates [69]. The consistency between SCQ, CARS and ADOS was reassuring. It may also indicate that the instruments indeed measure autism-specific traits as opposed to more general social difficulties or psychopathology. The unexpected finding that the number of autistic features was negatively related to age may be explained by the fact that the two children diagnosed with ASD were younger than 7 years. Finally, we decided to lower the SCQ cut-off score to 10 (versus 15) to include more participants with “autistic traits”.

The SDQ has been widely used in South Africa without adaptation, but validation studies are limited [36]. Mellins (2018) evaluated the factor structure and psychometric characteristics [70], while Sharp (2014) found the SDQ-Parent (versus teacher or self-report versions) showed the best construct validity in a Sesotho population [71]. The 90th percentile cut-off was initially suggested by Goodman to determine high-risk scores [72], and most studies have applied these UK cut-off values [44]. This has in many cases resulted in higher numbers of South African children with scores above the cut-off values. Nazareth and colleagues (2022) assessed a cohort of 6–8-year-old children from Kwazulu-Natal and found that thresholds applying the 90th centile cut-off values for his participants were higher than UK values (e.g. total score 23 versus 20 in the UK [73]. We opted to use the UK cut-off scores of “extremely high” which is the 95th centile cut-off, to limit numbers for deep phenotyping. In addition, we also included children whose parents reported any degree of negative impact of the reported behaviour on family functioning (impact score ≥ 1).

Our preliminary analyses indicated relatively low verbal and performance scores in this sample, as measured both on the MSEL and WASI. These findings are broadly consistent with previous South African research, including an adolescent sample from a similar socio-economic background [74] and an earlier study involving 600 SPS pre-schoolers that used the Kauffmann Assessment Battery for Children [32]. This suggests that our findings were not specific to the use of the MSEL and WASI instruments. However, the concern remained that biases of these instruments such as language usage or test-taking experience cannot be fully disentangled from “true” risk factors in cognitive development. Testers’ impressions were that despite scoring in this lower range, most of the children were coping at school and in daily living, and that this did not reflect their academic potential. It was hypothesised that the children’s adaptive behaviour or “street smartness” may exceed expectations from WASI/MSEL estimates. Hence, for feasibility reasons, the cut-off for the low WASI was adjusted to Full-Scale Intellectual Quotient (FSIQ), verbal comprehension index score (VI) or performance index (PI) of 60 or lower. We decided to add the Vineland Socialisation and Daily Living subscale to the broad phenotype visit to assess adaptive skills, in order to help refute or confirm whether children with very low WASI scores would indeed have “intellectual disability”.

One aim of the BONO study will be to explore the separate and cumulative effects of prenatal factors, ACEs, and level of education and family environment on children’s and adolescents’ cognitive development. Preliminary findings from the RPQ indicate that many children in our cohort had experienced a substantial number of adversities. Although findings from this feasibility study are limited as the instrument was only administered to less than half of the sample, these factors have been associated with developmental delays in Western HIC [75, 76].

Furthermore, although in our sample the correspondence between maternal and child WASI scores was lower than expected, maternal cognitive development is associated with maternal education, and higher maternal education has been in turn associated with more stimulating activities [77], learning environments and availability of learning materials such as books or toys [78]. To test this, our in-house RPQ measure includes a section on children’s daily activities, such as the nature and frequency of reading books to children, playing, storytelling, etc.

To summarise, the threshold (cut-off) scores for deep phenotyping were lowered for autistic features on the SCQ to 10 to include children with milder social-communication difficulties. However, to restrict the number of children invited for deep phenotyping based on internalising/externalising score or IQ, the cut-off on the SDQ total difficulties score was set to “extremely high” (> 95th centile) and the cut-off for the WASI FSIQ, verbal or performance subscales to ≤ 60.

Hence, in the design of the main study, participants will be sequentially selected; first, based on autistic traits (which may include children with *co-occurring* internalising/externalising symptoms and/or low WASI scores), second on internalising or externalising symptoms (which may include children with low WASI scores) and third based on low WASI scores (< 60). As a comparison group, (i) an equal number of randomly selected children will be selected to participate in the deep-phenotyping phase and (ii) a subset of children with full-scale WASI scores > 100 (*N* = 120) to explore potential protective factors. Together, we expect that 25–33% of children will be invited to participate in the deep-phenotyping phase.

### Adequate resources to manage and implement the study

Resources were available to comprehensively assess 2000 children on the study protocol comprising the broad and deep-phenotyping phases with a maximum of 3000 visits. After adjusting the cut-offs for externalising/internalising mental health features and developmental delay/intellectual disability, we thus restricted the number of children returning for the second deep-phenotyping visit to a maximum of 33%.

### Referral procedures

We initially met with the community psychiatric mental health and social services regarding the study, confirming capacity and a direct referral pathway. The research unit is situated in a tertiary healthcare facility, and this facilitated referrals to specialist clinics and the psychiatric emergency unit. All the research workers were familiarised with referral protocols for community health and educational services. A substantial proportion (21%) of children were referred for further evaluation and management, necessitating liaison with service providers. This suggests that although this was a research study, it led to direct clinical benefit for participants.

### Study limitations

We are aware of several limitations of the study protocol and this feasibility study. First, we did not have the time nor resources (nor was it the aim of this study) to formally validate clinical and experimental measures in this South African mixed-ancestry population. This may affect interpretation of results, such as FSIQ levels. Second, as we intended to acquire the same measures in all study participants (aged 4–16 years), we relied on parent-administered measures to assess clinical features, although discrepancies between self- and parent report notably for internalising behaviours are known [79]. Third, although the eldest children will turn 16 years over the course of the main study, we did not trial the measures in the 12–16-year age group. This was due to a delay in the start of the main study due to the COVID-19 pandemic. Fourth, the paucity of paternal information on cognitive and emotional measures was an additional limitation of the study as we did not recruit fathers unless they were the primary caregiver of the child. Fifth, the RPQ was only administered to 40% of the caregivers as additional ethical approval for use of the questionnaire was required with proposed referral strategies. In addition, due to misunderstandings, the SDQ “impact” factors were not collected on all participants. Sixth, some of the participant feedback was anecdotal and not systematically recorded. Finally, while we asked participants about their understanding and acceptability of the proposed measures, we did not involve people with lived experience in the study design itself.

## Conclusions

The feasibility study enabled the team to develop efficiency and expertise for the planned multimodal study. It demonstrated (1) high recruitment/retention capability, (2) suitability of procedures and high acquisition rates of most clinical and experimental measures, (3) acceptability of the visit duration to participants, (4) adaptability to select informed cut-off scores for deep phenotyping, (5) adequate resources for anticipated participant numbers and (6) the ability to create appropriate referral pathways for study participants needing further assessment and treatment. Preliminary findings showed very high internalising and externalising scores, a shift in the WASI and MSEL score distribution, yet a similar number of autistic cases as would broadly be expected from HIC prevalence rates, and very high rates of adverse childhood experiences. Procedures were iteratively refined and adapted according to participant and researcher feedback to inform the implementation phase of the main study going forward.

## Supplementary Information

Additional file 1. Description of Behavioural Questionnaires administered to Caregivers.

Additional file 2. Adaptations to Mullen Scales of Early Learning.

Additional file 3. Aims-2-Trials: inter-rater-reliability/interscorer reliability procedure: Safe Passage Cohort-South Africa.

Additional file 4. Classification of children with and without autism using Childhood Autism Rating Scale and Autism Diagnostic Observation Schedule.

## Data Availability

The datasets used and/or analysed during the current study are available from the corresponding author on reasonable request.

## Abbreviations

ACE: Adverse childhood experiences
ADOS-2: Autism Diagnostic Observation Schedule-Second Edition
AIMS-2: Autism Innovative Medicine Trials
ASD: Autism spectrum disorder
CBQ: Child behaviour questionnaire
CO-LIFE: Childhood Oxford-Liverpool Inventory of Feelings and Experiences
CRI-R: Childhood Routines Inventory-Revised
DAWBA: Development and Well-being Assessment
EEG: Electroencephalogram
ERP: Early receptor potential
EU-AIMS: European Union Autism Innovative Medicine Trials
FSIQ: Full-Scale Intelligence Quotient
HIC: High-income countries
LMIC: Low- and middle-income countries
MSEL: Mullen Scales of Early Learning
O-LIFE: Oxford-Liverpool Inventory of Feelings and Experiences
PIQ: Performance intelligence quotient
RPQ: Risks and Protective factors Questionnaire
SCQ: Social Communication Questionnaire
SDQ: Strengths and Difficulties Questionnaire
SEQ: Sensory Experience Questionnaire
SPS: Safe Passage Study
SRS-2: Social Responsiveness Scale-Second Edition
VIQ: Verbal intelligence quotient
WASI: Wechsler Abbreviated Scales of Intelligence-second edition
ZAR: South African Rands
