# Supplementary material for: Identifying biomarkers of neurodevelopmental and mental health outcomes in a prospective longitudinal cohort of South African children: design and feasibility of the Safe Passage BONO study
Source: Pilot Feasibility Stud. 2026 May 12;12:64. doi: 10.1186/s40814-026-01790-1 (PMC13162464; doi:10.1186/s40814-026-01790-1)
Supplement: Supplementary file 1 — Additional file 1. Description of Behavioural Questionnaires administered to Caregivers. [file 40814_2026_1790_MOESM1_ESM.docx]

**Appendix 1 Description of Behavioural Questionnaires administered to Caregivers**

*Social Communication Questionnaire (SCQ)(1)*

The SCQ is a screening instrument for autism that has been standardised and validated on a cohort of children on the autistic spectrum. It comprises 40 items and was designed as a questionnaire version of the Autism Diagnostic Interview-Revised.

*Strength and Difficulties Questionnaire (SDQ) Child and Adult Versions (2)–*

The SDQ is a 25-item parent-rated inventory designed as a behavioural screening measure to assess the occurrence of behaviours that have been associated with conduct problems, hyperactivity, emotional symptoms, peer problems, and pro-social behaviour in 4–16-year-olds. The adult self-report form includes the same 25 traits as the child version.

*Childhood Oxford-Liverpool Inventory of Feelings and Experiences (CO-LIFE) and (O-LIFE)(3)*

The O-LIFE(adults) and CO-LIFE (children) are dimensional measure of schizotypal and psychotic traits in the general population, and individuals with neurodevelopmental or neuropsychiatric disorders. The CO-LIFE is normed for children between 2-18 years and consists of three subscales, reflecting cognitive disorganisation/ impulsivity, sensory perception/ magical thinking and social anxiety/ withdrawal. Here, only the 14 subscales sensory perception/ magical thinking subscale will be used.

*Childhood Routines Inventory-Revised (CRI-R)(4)*

The CRI-R is a parent-informed questionnaire adapted from the Childhood Routines Inventory (CRI) that assesses broader repetitive behaviours and restricted interests seen across a host of neurodevelopmental disorders, including stereotypies, motor and phonic tics, self-directed behaviours (“nervous habits”), insistence on sameness, and sensory sensitivities.

*Sensory Experience Questionnaire (SEQ)(5)*

The SEQ is able to characterize sensory features in young children with autism and differentiate their sensory patterns from comparison groups.

*Child Behavior Questionnaire (CBQ)(6)*

The CBQ uses caregiver reports to provide a detailed profile of young children's temperament and assesses intrapersonal and interpersonal competencies.

*Development and Well-being Assessment Questionnaire (DAWBA)(7)*

The Development and Well-Being Assessment (DAWBA) is a package of questionnaires, interviews, and rating techniques designed to generate ICD-10 and DSM-IV psychiatric diagnoses on 5-16-year-olds. Non-clinical interviewers administer a structured interview to parents about psychiatric symptoms in their child and resultant impact on the family.

References

1. Rutter M, Bailey A, Lord C. The Social Communication Questionnaire (SCQ) [Manual]. Torrance, CA: Western Psychological Services; 2003.

2. Goodman R. The strengths and difficulties questionnaire: A research note. J Psychol Psychiatry. 1997;38:581–6.

3. Evans DW, Lusk LG, Slane MM, Michael AM, Myers SM, Uljarević M, et al. Dimensional assessment of schizotypal, psychotic, and other psychiatric traits in children and their parents: development and validation of the Childhood Oxford-Liverpool Inventory of Feelings and Experiences on a representative US sample. J child Psychol psychiatry. 2018;59(5):574–85.

4. Evans DW, Uljarević M, Lusk LG, Loth E, Frazier T. Development of Two Dimensional Measures of Restricted and Repetitive Behavior in Parents and Children. J Am Acad Child Adolesc Psychiatry. 2017;56(1):51–8.

5. Baranek GT, David FJ, Poe MD, Stone WL, Watson LR. Sensory Experiences Questionnaire: Discriminating sensory features in young children with autism, developmental delays, and typical development. J Child Psychol Psychiatry Allied Discip. 2006;47(6):591–601.

6. Rothbart MK, Ahadi SA, Hershey KL, Fisher P. Investigations of temperament at three to seven years: the Children’s Behavior Questionnaire. Child Dev. 2001;72(5):1394–408.

7. Goodman R, Ford T, Simmons H, Gatward R, Meltzer H. Using the Strengths and Difficulties Questionnaire (SDQ) to screen for child psychiatric disorders in a community sample. Int Rev Psychiatry. 2003;15(1–2):166–72.
