# Supplementary material for: Identifying biomarkers of neurodevelopmental and mental health outcomes in a prospective longitudinal cohort of South African children: design and feasibility of the Safe Passage BONO study
Source: Pilot Feasibility Stud. 2026 May 12;12:64. doi: 10.1186/s40814-026-01790-1 (PMC13162464; doi:10.1186/s40814-026-01790-1)
Supplement: Supplementary file 2 — Additional file 2. Adaptations to Mullen Scales of Early Learning. [file 40814_2026_1790_MOESM2_ESM.docx]

APPENDIX 2

Adaptations to Mullen Scales of Early Learning

24. Verbal Analogies

A house is made of wood, a tent is made of …… (cloth, canvas, vinyl): children in this community were not familiar with tents, they rarely go camping, but they often reported making a “tent out of blankets” when they went to the beach, so we accepted “blankets” as an answer

26. Oral Vocabulary

Dime substituted with coin

Faucet substituted with tap

Canoe substituted with boat

27. Practical reasoning

In response to the question ‘What do you do when it starts to get dark?’ we accepted the following responses from the children “we buy / put in electricity/ we go to sleep/we light candles”.

28 Repeating Sentences II

3) we replaced “In the winter we go sliding down the hill on our sled” with “In the summer we go to swim in the sea”

Adaptations to the Wechsler Abbreviated Scale of Intelligence (WASI-2)

Word Substitutions

“candle’ instead of lamp

“crocodile” instead of alligator

“host” for entertain to avoid colloquialism

“onthaal” instead of vermaak to avoid colloquialism.

“almanac”(Afrikaans), calendar (English).

Picture Substitution

Pansy shell picture was replaced with a cockle shell (more familiar)
