# Supplementary material for: Identifying biomarkers of neurodevelopmental and mental health outcomes in a prospective longitudinal cohort of South African children: design and feasibility of the Safe Passage BONO study
Source: Pilot Feasibility Stud. 2026 May 12;12:64. doi: 10.1186/s40814-026-01790-1 (PMC13162464; doi:10.1186/s40814-026-01790-1)
Supplement: Supplementary file 3 — Additional file 3. Aims-2-Trials: inter-rater-reliability/interscorer reliability procedure: Safe Passage Cohort-South Africa. [file 40814_2026_1790_MOESM3_ESM.docx]

**Aims-2-Trials: inter-rater-reliability /interscorer reliability procedure: Safe Passage Cohort-South Africa**

In the interscorer reliability method as indicated in the Mullen Scale of Early Learning Manual, one tester administered the tests to a child, each evaluator scored the test independently. Testers alternated roles of tester/scorer and scorer. We used this method to obtain the following results:

**Mullen: Participant 1:**

| **Subtest Raw Scores** | **Tester 1** | **Tester 2** | **Agreement** |
| --- | --- | --- | --- |
| Visual Reception | 44 | 44 | Yes |
| Receptive Language | 46 | 46 | Yes |
| Expressive Lang | 45 | 45 | Yes |
|  |  |  |  |

**Participant 2:**

| **Subtest Raw Scores** | **Tester 1** | **Tester 2** | **Agreement** |
| --- | --- | --- | --- |
| Visual Reception | 47 | 47 | Yes |
| Receptive Language | 42 | 43 | No |
| Expressive Lang | 48 | 48 | Yes |
|  |  |  |  |

**Participant: 3**

| **Subtest Raw Scores** | **Tester 1** | **Tester 2** | **Agreement** |
| --- | --- | --- | --- |
| Visual Reception | 42 | 42 | Yes |
| Receptive Language | 37 | 37 | Yes |
| Expressive Lang | 41 | 41 | Yes |
|  |  |  |  |

The same procedure was conducted for the WASI-II. The results are as follows:

**WASI-II Participant: 4**

| **Subtest Raw Scores** | **Tester 1** | **Tester 2** | **Agreement** |
| --- | --- | --- | --- |
| Block Design | 21 | 21 | Yes |
| Vocabulary | 16 | 16 | Yes |
| Matrix Reasoning | 18 | 18 | Yes |
| Similarities | 17 | 17 | Yes |
| **Composite scores** |  |  |  |
| VCI | 78 | 78 | Yes |
| PRI | 100 | 100 | Yes |
| FSIQ-4 | 87 | 87 | Yes |
| FSIQ-2 | 88 | 88 | Yes |
