# Supplementary material for: Identifying biomarkers of neurodevelopmental and mental health outcomes in a prospective longitudinal cohort of South African children: design and feasibility of the Safe Passage BONO study
Source: Pilot Feasibility Stud. 2026 May 12;12:64. doi: 10.1186/s40814-026-01790-1 (PMC13162464; doi:10.1186/s40814-026-01790-1)
Supplement: Supplementary file 4 — Additional file 4. Classification of children with and without autism using Childhood Autism Rating Scale and Autism Diagnostic Observation Schedule. [file 40814_2026_1790_MOESM4_ESM.docx]

| Classification of children with and without autism using Childhood Autism Rating Scale and Autism Diagnostic Observation Schedule | | | | | | | |
| --- | --- | --- | --- | --- | --- | --- | --- |
| **Participant** | **Age** | **Sex** | **CARS Total Raw Score** | **CARS Classifications** | **ADOS Module** | **ADOS**  **Total score** | **ADOS Classification** |
| 1 | 4 years | Male | 16.5 | **nonautistic**  | 1 | 6 | **nonautistic** |
| *2 | 4 years | Male | 31.5 | **autism**  **mild to moderate** | 1 | 19 | **autism** |
| 3 | 6 years | Male | 21,5  | **nonautistic** | 2 | 6 | **nonautistic** |
| *4 | 5 years | Male | 28 | **nonautistic** | 2 | 14 | **autism spectrum** |
| 5 | 6 years | Male | 18 | **nonautistic** | 1 | 7 | **nonautistic** |



*Child diagnosed with autism/autism spectrum in a developmental clinic
